# A qualitative evidence synthesis (QES) exploring the barriers and facilitators to screening in emergency departments using the theoretical domains framework

**DOI:** 10.1186/s12913-023-10027-3

**Published:** 2023-10-11

**Authors:** Louise Barry, Sylvia Murphy Tighe, Anne Griffin, Damien Ryan, Margaret O’Connor, Christine Fitzgerald, Siobhan Egan, Rose Galvin, Pauline Meskell

**Affiliations:** 1 Department of Nursing and Midwifery, Faculty of Education and Health Sciences, University of Limerick, Castletroy, Limerick, Ireland; 2 Ageing Research Centre, Health Research Institute, University of Limerick, Limerick, Ireland; 3 School of Allied Health, Faculty of Education and Health Sciences, University of Limerick, Castletroy, Limerick, Ireland; 4 Emergency Department, University Hospital Limerick, Dooradoyle, Limerick, Ireland; 5 Department of Ageing and Therapeutics, University Hospital Limerick, Dooradoyle, Limerick, Ireland; 6 Clinical Research Support Unit, University Hospital Limerick, Dooradoyle, Limerick, Ireland

**Keywords:** Barriers and facilitators, Emergency care settings, Screening, Screening tools, Qualitative evidence synthesis, Stakeholder experience, “Best Fit” framework synthesis

## Abstract

**Background:**

Validated screening tools can be utilised to detect early disease processes and risk factors for disease and adverse outcomes. Consequently, identifying individuals in need of early intervention and targeted assessment can be achieved through the implementation of screening in the ED. Successful implementation can be impacted by a lack of resources and ineffective integration of screening into the clinical workflow. Tailored implementation processes and staff training, which are contextually specific to the ED setting, are facilitators to effective implementation. This review will assist in the identification of barriers and facilitators to screening in the ED using a QES to underpin implementation processes. Healthcare workers engage in screening in the ED routinely. Consequently, this review focused on synthesizing the experience of healthcare workers (HCWs) who are involved in this process. This synthesis is informed by a QES protocol published by the lead author in 2021 (Barry et al., HRB Open Res 3:50, 2021).

**Methodology:**

A comprehensive literature search, inclusive of grey literature sources, was undertaken. Initially, an a priori framework of themes was formed to facilitate the interpretation and organisation of search results. A context specific conceptual model was then formulated using “Best fit” framework synthesis which further assisted in the interpretation of data that was extracted from relevant studies. Dual blind screening of search results was undertaken using RAYYAN as a platform. Thirty studies were identified that met the inclusion criteria. Dual appraisal of full text articles was undertaken using CASP, GRADE CERQual assessed confidence of findings and data extraction was performed by two reviewers collaboratively.

**Findings:**

This is the first known synthesis of qualitative research on HCW’s experiences of screening in the ED. Predominantly, the findings illustrate that staff experience screening in the ED as a complex challenging process. The barriers and facilitators identified can be broadly categorised under preconditions to screen, motivations to screen and knowledge and skills to screen. Competing interests in the ED, environmental stressors such as overcrowding and an organisational culture that resists screening were clear barriers. Adequate resources and tailored education to underpin the screening process were clear facilitators.

**Trial registration:**

PROSPERO: CRD42020188712 05/07/20.

**Supplementary Information:**

The online version contains supplementary material available at 10.1186/s12913-023-10027-3.

## Introduction

Many individuals with chronic health conditions have a high risk of healthcare usage due to complex healthcare needs (Marcoux et al., 2017 [21]). To adapt and meet these needs, early identification and intervention of those at risk is vital (Marcoux et al., 2017 [21]) Ideally, screening tools identify patients early enough to provide treatment and avoid or reduce symptoms and other consequences, improving health outcomes of the population at a reasonable cost (Iragorri & Spackman, 2018 [14]; Weber et al., 2023 [32]). In clinical settings, this screening process supports timely referral for “at-risk” or vulnerable groups and specialised intervention to reduce the risk of adverse outcomes and disease progression (Marcoux et al., 2018 [21]; Iragorri & Spackman, 2018 [14]).

For many patients, the Emergency Department (ED) is a critical access point for healthcare services particularly among those with limited access to resources (Weber et al., 2023 [32]). ED screening can identify those who need specialist referral or intervention and consequently serve as a contributor to individual and population health (Weber et al., 2023 [32]). In addition, ED screening initiatives should focus on evidence-based strategies and take local epidemiology, ED capacity, financial sustainability and collaboration with community services into consideration to ensure successful implementation (Weber et al., 2023 [32]).

Internationally, screening tools to identify the risk of frailty, sepsis, falls, functional decline and healthcare utilisation have been implemented in EDs to underpin the ever-increasing focus on preventative medicine (Kirk et al., 2016 [17]; Marcoux et al., 2018 [21]). Screening tools have been developed for ED patients to help detect multiple diseases and risk factors, ranging from nutrition status to sepsis to suicide risk (Mullinax et al., 2018 [26]; Filbin et al., 2018 [11]). However, these screening tools can vary in complexity, resources required to administer and time needed to complete (Kirk et al., 2016 [17]; Asomaning &Loftus, 2014 [1]). Therefore, in a busy ED with time pressures and a lack of available resources to underpin complex screening processes, the implementation of screening tools can be challenging (Asomaning & Loftus, 2014 [1]). New practices within this setting has also proved problematic due to perceived irrelevance of screening this environment, practice demands, time pressures and a high level of stress and unpredictability (Asomaning & Loftus, 2014 [1]; Creswick et al., 2009 [6]; Tavender et al., 2014 [31]). Thankfully, screening is recognised in acute settings as being clinically important, however, it is often overlooked or not prioritised due to heavy workloads, competing demands and a lack of time (Eagles et al. 2022 [10]; Liu et al., 2022 [20]). Uptake is also likely impacted by competing interests and priorities and ease of use in a busy ED environment. Furthermore, screening tools in the ED are often integrated within care bundles, pathways and protocols and this must be considered when planning implementation. Overall, successful implementation is dependent on pre-implementation adaptation, testing and staff education (McCusker et al., 2007 [23]).

ED staff are focused on flow culture which can act as a barrier (Kirk and Nilsen, 2016 [16]). In the ED, screening tools which do not support the flow of patients are met with resistance by staff (Kirk and Nilsen, 2016 [16]). To ensure systematic screening, ED staff uptake and ensure optimal implementation of screening, local barriers and facilitators need to be identified and explored (Kirk et al., 2016 [17]). ED staffs’ professional roles, responsibilities, identity, actions and senses making is impacted and moulded by the local culture and consequently provides staff with differing perceptions and experiences of specific barriers and facilitators (Kirk et al., 2016 [17]). Prior to implementation of screening tools, there must be an in-depth understanding of local culture and how new tools make sense within the cultural context (Kirk et al., 2016 [17]).

However, although population health initiatives encourage a broader perspective on ED visits, screening should not detract from the primary purpose of the ED: management of acute illness and injury (Weber et al., 2023 [32]). Therefore, screening that results in longer wait times increased length of stay, or other adverse effects on patient care must be avoided. Those implementing screening initiatives should consider that local capacity should always dictate whether screening is feasible or prudent given the demands of screening and the resources available (Weber et al., 2023 [32]). Findings elsewhere suggest that a multidisciplinary approach is vital when implementing screening in the ED and requires multifaceted interventions including education, documentation changes and consistent communication and teamwork (Martin et al., 2022 [22]; Tavender et al., 2014 [31]). A multidisciplinary approach to screening is advocated with the integration of screening based on local capacity, triage processes and local ED pathways and resources.

Furthermore, the burden placed on clinical staff in the ED and modifications to the ED workflow must be considered to reduce any effect on clinical staff (Weber et al., 2023 [32]). Clinical staff in the ED often faces mismatched patient/staff ratios, boarding of admitted patients, and overcrowding. Adding numerous screening questions can detract from their care of emergency conditions (Weber et al., 2023 [32]). Therefore, in terms of the timing of screening and those administering relevant tools, this is largely dependent on contextual factors which must be considered prior to implementation. However, in an often chaotic and high-pressure ED environment, accuracy, while ensuring brevity is vital (Weiner et al., 2019 [33]). Screening should be undertaken early in the ED admission to ensure appropriate interventions and referral to specialist services if warranted (Weber et al., 2023 [32]). Therefore, screening in triage is often employed to ensure that patients are referred promptly.

The barriers and facilitators to screening in the ED has been explored from the perspective of healthcare workers in numerous studies (Tavender et al., 2014 [31]; Kirk et al., 2016 [17]; Eagles et al. 2022 [10]; Liu et al., 2022 [20]). The identification of these factors is vital to ensure successful implementation and consequently attain appropriate healthcare for those at risk. A gap exists to synthesize the findings from these studies to give further insight into the professional and organisational barriers and facilitators to screening. Furthermore, there is a lack of previous reviews that succinctly identify factors which impact on screening implementation. As illustrated, although screening tools differ in terms of approach and utilisation, those employed in the ED are impacted by similar barriers and facilitators to implementation. This hypothesis has yet to be clarified. Furthermore, this review was undertaken to inform the implementation of adult screening for risk in the ED, therefore this was the focus of the review. To give broader insight into the barriers and facilitators to screening in the ED, this will be inclusive of adult screening and multiple screening methods. The review question is defined as “*What are the barriers and facilitators to adult screening in the ED*? 

## Methods

This qualitative evidence synthesis (QES), adopted the PRISMA Statement (Preferred Reporting Items for Systematic Reviews and Meta-Analyses) flow diagram (Page et al., 2021 [29]) and used “best-fit” framework synthesis (BFFS) (Booth and Carroll, 2015 [4]) to produce a context specific conceptual model to explain and describe the barriers and facilitators to screening in emergency departments. A detailed protocol for this review was published (Barry et al., 2021 [3]) and registered with PROSPERO (CRD42020188712 05/07/20).

### Inclusion and exclusion criteria 

Studies that pertained to the assessment/screening of adults (> 18 years) were considered. The studies included explicitly discussed factors that impacted on screening or the implementation of screening within the ED from the perspective of healthcare workers. Qualitative studies utilising qualitative methods of data collection and analysis were considered, mixed method studies that included a qualitative component utilising qualitative methods of data collection and analysis was also considered suitable for inclusion. See Table 1.

**Table 1:** Inclusion/Exclusion Criteria

Criteria for selection	Included	Excluded
**Types of article**	Primary research studies	Descriptive articles, literature reviews, systematic reviews, QES and integrative reviews
**Types of studies**	Qualitative studies utilising qualitative methods of data collection and analysis Mixed method studies that include qualitative component utilising qualitative methods of data collection and analysis Theses utilising qualitative methods of data collection and analysis	Quantitative research Qualitative components of mixed methods studies that do not have distinct qualitative methods of data collection and analysis • Studies which pertain to paediatric screening or screening for domestic violence • Studies which describe screening for mental health disorders or suicide risk • Studies which pertain to triage screening or categorisation
**Types of participants**	Health care workers: Professionals (Doctors, nurses, midwives, allied health professionals, pharmacists, administrative staff)	Informal carers/family members Health care staff who do not have direct patient contact (Laboratory staff)
**Types of settings**	Emergency departments, acute assessment units (MAU, SAU, AAU)	General wards Non-workplace setting
**Types of outcomes**	Barriers and facilitators to screening/assessment/triage of adults > 18yrs and that fit under the Theoretical Domains Framework (TDF)	

### Search strategy

**Fig. 1:**
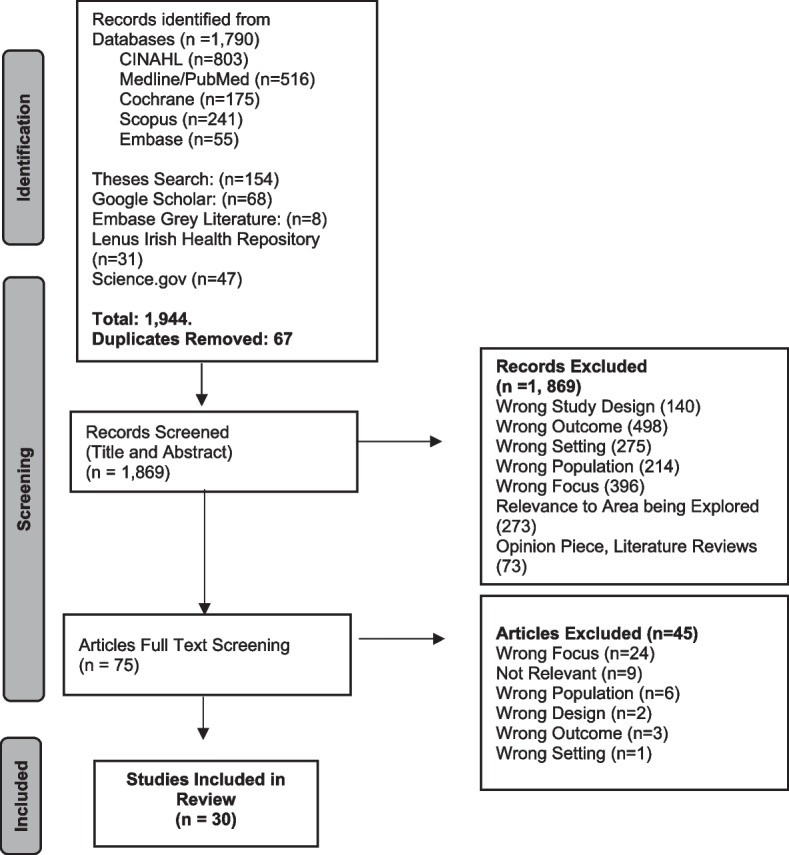
The PRISMA flow diagram

The initial search was conducted in April of 2020 and updated in April of 2022. The search was focused to sources published in the last 10 years (2010–2022) to reflect current screening approaches in the ED. No other limitations to the search were applied. Scopus, Medline Ebsco, Embase, Pubmed, CINAHL Ebsco, and Cochrane were systematically searched. Open Grey, Google Scholar, Lenus Irish Health Repository Science. Gov and Embase Grey Literature Sources were searched to identify relevant theses. Keyword searching of electronic databases was undertaken. Medical subject headings (MeSH) and specific database headings were also used to further identify relevant search terms. Truncation of key terms was used to broaden the search and ensure that all appropriate key words were utilised. Please see the detailed protocol for this review (Barry et al., 2021 [3]) which had greater detail pertaining to the development of the search strategy. Supplementary file 1 contains the Medline search string and Fig. 1 contains the PRISMA Flow Diagram.

### Relevance appraisal

LB and PM performed title and abstract screening independently and in duplicate, initially database search results were uploaded to Endnote where duplicates were removed and then merged into RAYYAN. RAYYAN facilitated the blind assessment of results to limit bias (Ouzzani et al., 2016 [27]). Full text screening of remaining articles was also performed by LB and PM against the relevant inclusion/exclusion criteria. Where conflicts arose, a consensus meeting was convened and in instances where a consensus couldn’t be reached, RG acted as a third independent reviewer. Consensus was reached among LB and PM with RG convening to resolve conflicts pertaining to three articles. A record of rationale for exclusion was kept for all studies where the full text of the article was retrieved, and the paper was subsequently excluded Supplementary file 2.

### Quality appraisal (see Supplementary file 3)

The quality of included studies was then assessed using the Critical Appraisal Skills Programme (CASP) (CASP, 2018 [5]). This assessed if studies had a clear and rigorous methodology, findings that were supported by evidence, clear evidence of researcher reflexivity and clear consideration of ethical principles. The appraisal was carried out independently by two reviewers LB and RG. Any conflicts were discussed until consensus was achieved. A third author (PM) was available to mediate any conflicts, but this was not required.

### Data extraction and synthesis

Initially, to assist in the organisation and interpretation of descriptive study characteristics, data was extracted into a table of articles which was number to correspond with each article (1–30) Supplementary file 4. Best-Fit Framework Synthesis (BFFS) produces conceptual models to assist in explaining and describing the health behaviours or decision-making of patients or other groups by using a transparent and pragmatic process (Dixon-Woods, 2011 [9]). To facilitate the synthesis of primary research, using this method, an a priori framework was identified to facilitate the evidence synthesis. This a priori framework was based on the definitions and component constructs of the Theoretical Domains Framework (TDF) outlined by Atkins et al. (2017) [2] Supplementary file 5. Implementation researchers and behavioural scientists developed the TDF framework to identify theories relevant to implementation (Atkins et al., 2017 [2]). There are clear advantages to utilising this framework as it can form the theoretical basis for implementation studies, provide a strong rationale for potential reasons for slow diffusion of evidence into practice and a clear methodology to progress from theory-based investigation to intervention (Atkins et al., 2017 [2]). Furthermore, the TDF can facilitate comprehensive assessment of behavioural determinants in qualitative studies and assist in interpretation of rich datasets (McGowan et al., 2020 [24]). Furthermore, behaviour change is required of those involved in the screening process and this is facilitated by understanding the determinants of behaviours, in this instance, Barriers and Facilitators. The TDF facilitates the interpretation of study findings by organising them into constructs which rationalise these behaviours and express them under relevant domains. Quotes and excerpts from relevant articles were categorised under domains by classifying them under constructs. This process of coding the data is illustrated in Supplementary file 6. Two researchers examined the coded data under the TDF domains (LB and PM). Using a template on google forms, data were extracted from the discussion and results sections of included studies directly into the TDF a priori framework.

https://docs.google.com/forms/d/1G4JxueDpcyy0B2Qv_31RYS5Ce6AmrCFoyj9sbnkSV-4/edit#responses**(**Supplementary Link).

Consequently, 11 findings with associated barriers and facilitators were identified under relevant constructs within each domain. This process was deductive and included themes or categories of findings, primary data extracts and author commentary and interpretations about the data collected Supplementary file 7. Concepts from the TDF were then clustered and synthesised to form a final set of themes representing the whole dataset Supplementary file 8. Therefore, after extracting data from studies pertaining to healthcare workers experience of screening in the ED, barriers and facilitators were identified from the constructs. The interpretative work to identify relationships between domains and their associated component constructs was conducted collaboratively (LB, RG and PM) with all stages of the analysis discussed by the review team until there was broad agreement. This will be further discussed under the discussion of findings.

### Applying GRADE CERQual

The Grading of Recommendations Assessment, Development and Evaluation (GRADE) Confidence in Evidence from Reviews of Qualitative Research (CERQual) approach was used to enhance transparency and confidence in reporting of QES findings. The contribution of lower quality papers, to the formation of themes, were assessed in conjunction with that of findings from higher quality studies. The Methodological Limitations, Coherence, Relevance and Adequacy of findings were assessed (Lewin et al., 2015 [19]). The CASP appraisal tool was utilised to assess for possible methodological limitations. The overall coherence and relevance of individual review findings was also assessed. This ensures that major themes and sub-themes are relevant to the overall review aim and are grounded in the data from included studies (McGrath, 2019 [25]). To ensure adequacy, the richness and quantity or review findings was also assessed. This has considered the strengths and weaknesses of studies and the number of studies which informed the findings. This yielded an overall assessment of confidence in review findings.

The GRADE CERQual process was undertaken by LB, AG and SE and reviewed by PM where areas of concern were pin pointed. Each phase was discussed by the reviewers and conflict resolution was attained through discussion and consensus.

### GRADE CERQual assessment criteria

GRADE, Grading of Recommendations Assessment, Development and Evaluation; CERQual, Confidence in Evidence from Reviews of Qualitative Research; CASP, Critical Appraisal Skills Programme (Lewin et al., 2015 [19].

**Table:** 

GRADE CERQual Component	Rationale/Concerns
Methodological Limitations (CASP)	The primary studies underlying a review finding are shown to have problems in the way they are designed or conducted
Coherence	We are less confident that the finding reflects the phenomenon of interest when: • Some of the data contradicts the findings • Some of the data is ambiguous
Adequacy	The data underlying a review finding are not sufficiently rich or only come from a small number of studies or participants
Relevance	The contexts of primary studies underlying a review finding are substantively different from the context of the review aim/question

### Findings

The search identified 1,944 articles. A total of 75 full-text articles were reviewed independently and a consensus was reached to include 30 articles. Overall, eleven screening modalities were included, and all were used to detect the risk of adverse outcomes/ functional decline, risk or presence of serious illness or to aid diagnostics and treatment plans. 
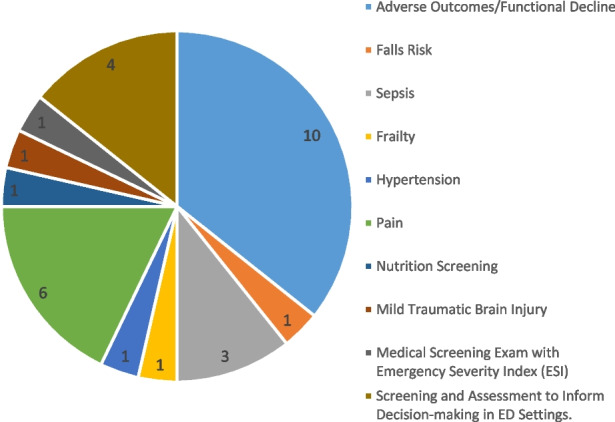


The studies were conducted in Australia (*n* = 7), UK (*n* = 6), USA (*n* = 6), Denmark (*n* = 2) Canada (*n* = 3) Canada/Ethiopia Partnership (*n* = 1), Brazil (*n* = 1), United Arab Emirates (*n* = 1), Italy (*n* = 1), Netherlands (*n* = 1) and Sweden (*n* = 1).

A qualitative descriptive design was the most common methodology employed (*n* = 12). Mixed methods studies inclusive of a qualitative aspect accounted for 5 of the studies included.
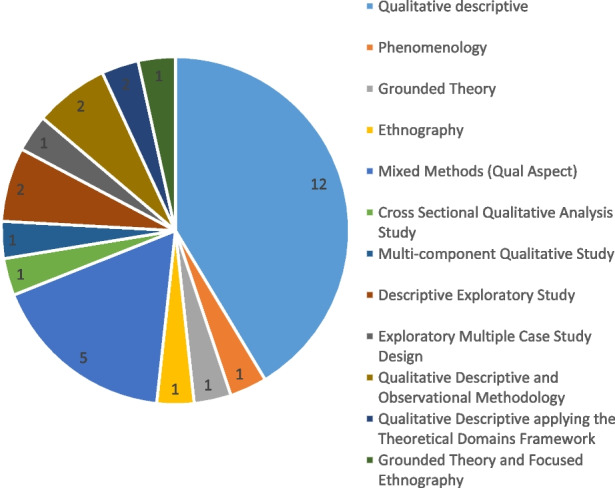


Twenty-three studies were based in the ED alone and seven studies were based in a combination of acute settings linked with the ED, either by the service or pathway provided e.g. Medical Assessment Unit (MAU) or Surgical Assessment Unit (SAU). Therefore, these settings were inextricably linked e.g. Stroke intervention team that work out of the ED.
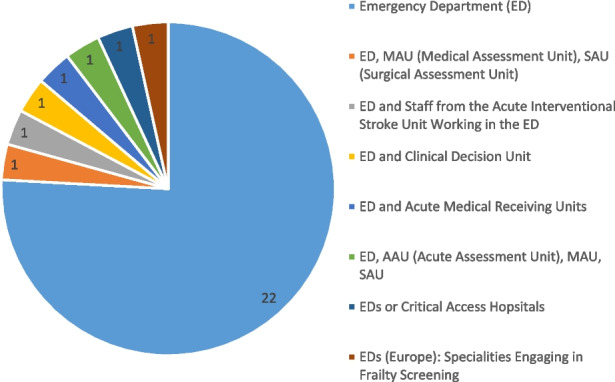


Predominantly nurses (*n* = 30 studies), and doctors (*n* = 18 studies) who worked in the ED or had ED experience were interviewed or observed. Physiotherapists participated in four studies, pharmacists in three and occupational therapists and speech and language therapists in two. Other disciplines were represented to a far lesser extent and featured in only one study each e.g. Hospital Administrators, ED Managers and Directors and Physician Assistants. Weber et al. (2023 [32]).
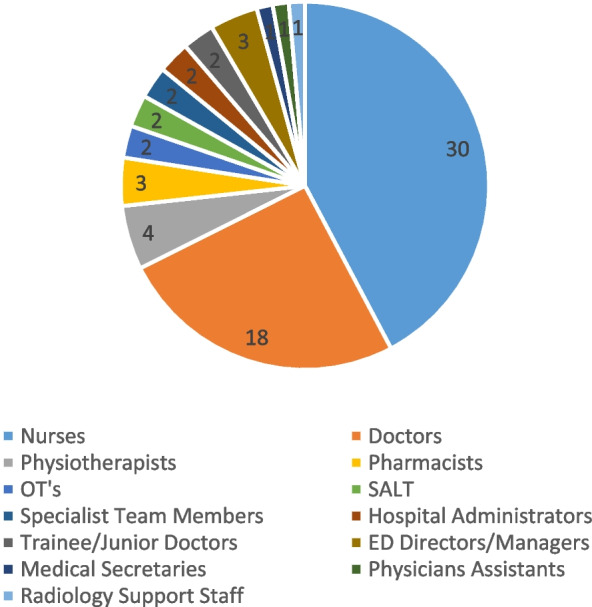


### Statement of principal findings

Upon CASP appraisal, no significant concerns were noted for 15 articles (High Quality), 10 articles were deemed as having minor methodological limitations, one study as having minor-moderate limitations and two as having moderate (Moderate Quality). Only two studies were deemed as having major limitations (Low Quality). Upon application of GRADE CERQual, a high level of confidence in research findings was attained with minor concerns pertaining to adequacy and methodological limitations overall. A large number of studies from diverse populations and settings with predominantly rich data informed review findings. This process is detailed in Supplementary file 9. Overall, HCWs are motivated and empowered to engage in screening if involved in the implementation process and offered education and tailored supports and incentives to sustain screening practices in an often-challenging environment. Professional confidence impacted the screening process where staff who felt competent and skilled to screen facilitated the process and were empowered to maintain screening practices. This empowerment originated from HCWs also performed screening for several reasons including commitment to the patient and maintaining patient safety, these were key motivators for staff to maintain screening. ED staff were keen to develop skills to engage in screening and referral process competently and these skills were honed through practice-based experience, educational opportunities and skills assessment. On initial involvement in the screening process, HCWs felt that screening was a simple task but as they progressed in their role learned the complexity of the screening process became more evident as it involved multiple staff members and interdependent tasks. Consequently, screening was often perceived by staff as complex with multiple challenges such as time, resources, lack of supports and ever-increasing workloads.

## Discussion of findings

**Fig. 2:**
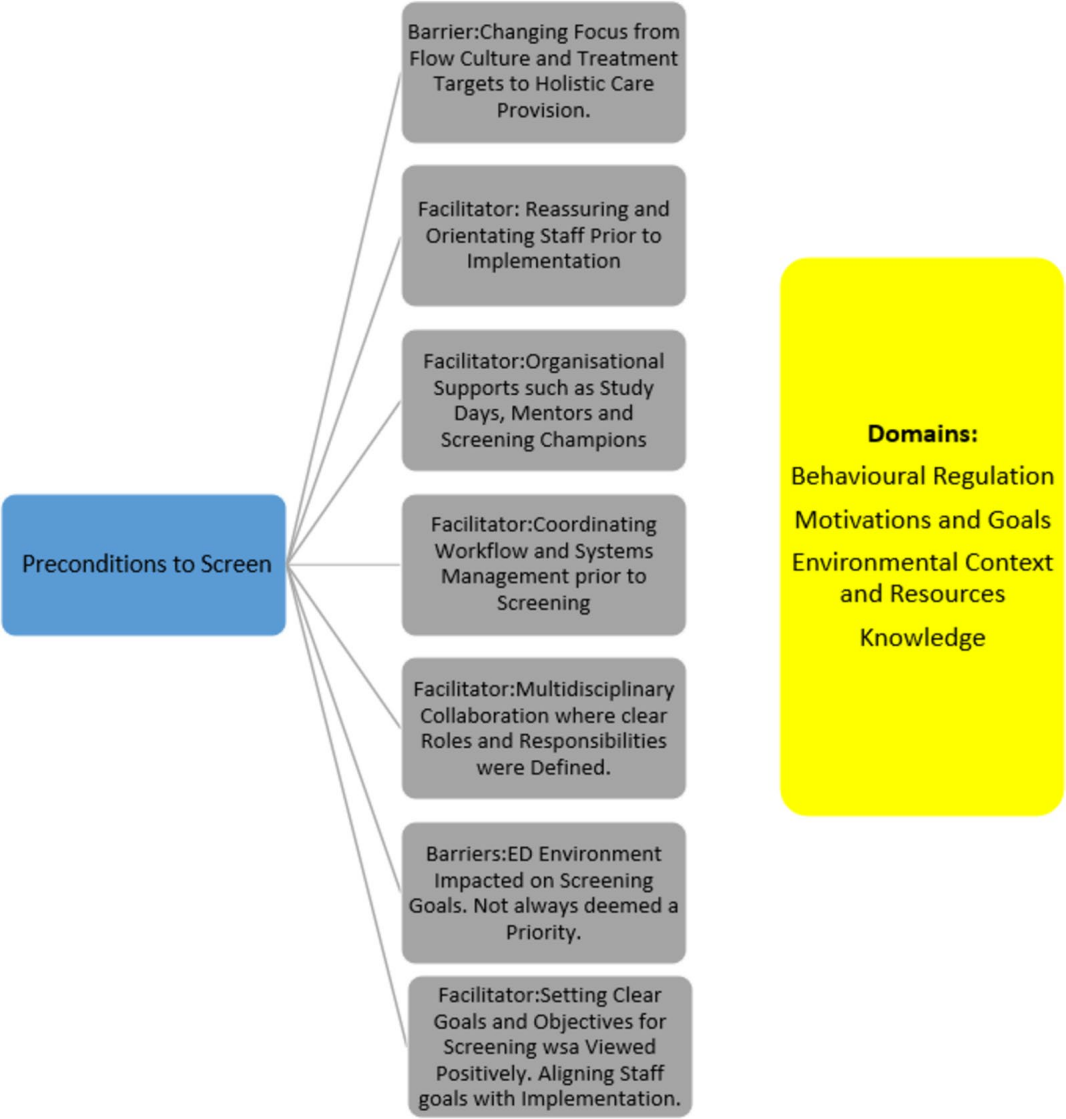
Five distinct facilitators and two barriers were identified from the theoretical domains synthesis that were preconditions to screening

**Fig. 3:**
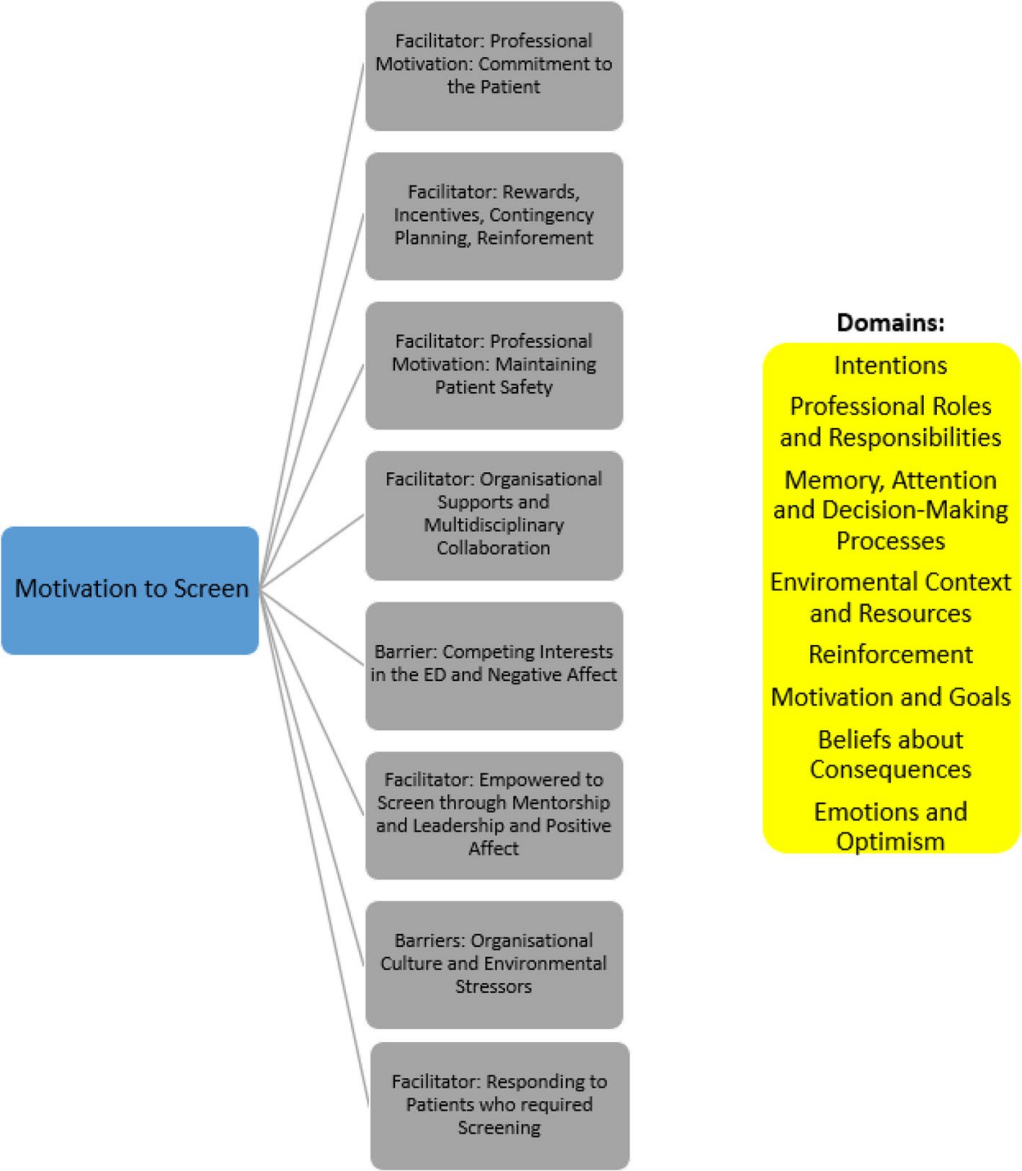
Six distinct facilitators and two barriers were identified from the theoretical domains synthesis that pertained to motivations for screening

**Fig. 4:**
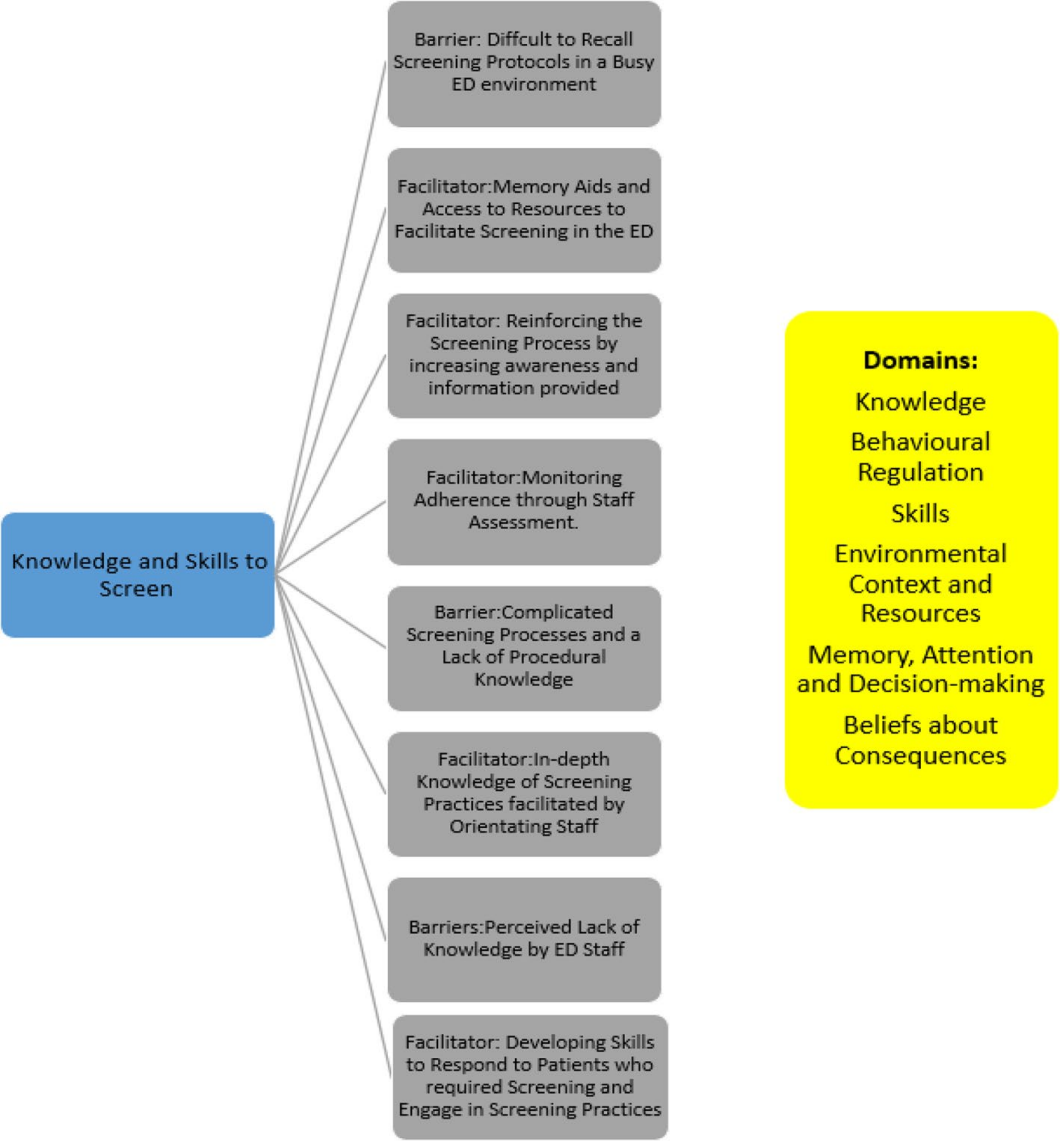
Five distinct facilitators and three barriers were identified from the theoretical domains synthesis that pertained to required knowledge and skills to screen

This evidence synthesis is the first known study to synthesise all available qualitative research on HCW’s views of experiences of screening in the ED. This review identified 30 studies that met the inclusion criteria. The results show with high confidence (based on the GRADE CERQual) that staff find screening in ED a complex challenging process. Consideration of the issues around screening can be broadly categorised under preconditions to screen, motivations to screen and knowledge and skills to screen. This was achieved by following a similar process to Ojo et al. (2019 [28]) where barriers and facilitators were deductively mapped to TDF domains and then further categorised under themes. Supplementary file 7. The process of how of these findings were mapped via domains and data mapped to constructs is included in Fig. 2, 3 and 4. Overall, competing demands in the ED complicated the process of screening for staff. These findings complement the current evidence base (Asomaning and Loftus, 2014 [1]; Liu et al., 2022 [20]) but provide greater insight into the experience of ED staff. To further complicate ED staffs’ involvement in screening, competing interests pertaining to the care of the acutely ill patient impacted on their adherence. A lack of time, the prioritisation of other patient care needs and insufficient targeted education related to screening tools and processes are commonly recognised as distinct barriers to screening in the ED (Eagles, 2022 [10]; Kirk, 2016 [17], Weber et al., 2023 [32]) and the QES findings reflect this.

### Preconditions to screen 

Evidence from this review highlights the importance of a number of preconditions for screening to be met to optimise effectiveness. This finding reflects the challenge of maintaining screening practices in the shadow of an increased pressure of ED’s to manage growing numbers of patients with increasing co-morbidities, in a setting where patients can rapidly deteriorate. Findings elsewhere suggest that a multidisciplinary approach is vital when implementing screening in the ED and requires multifaceted interventions including education, documentation changes and consistent communication and teamwork (Martin et al., 2022 [22]; Tavender et al., 2015 [31]). This synthesis supports this finding but further identified that organisational coordination must facilitate the screening process with supports such as study days and the appointment of mentors and champions to empower staff a priority. The coordination of workflow and systems management to facilitate screening must also be a priority at managerial and organisational level. In agreement with Martin et al. (2022) [22] who implemented delirium screening, evidence within this review identifies that screening processes must be developed at a multidisciplinary level collaboratively to ensure buy-in from staff. Furthermore, the impact of the ED environment must be mitigated at an organisational level to ensure that those on the frontline can engage in screening without a significant impact on their workload or well-being. Kirk and Nilsen, (2016) [16] highlighted that a focus on flow culture can be a barrier to implementing screening. Similarly, this synthesis found that the change of focus from treatment targets and flow culture to the provision of holistic care via screening must also be emphasised to ensure screening prioritisation.

### Motivation to screen

To successfully implement and maintain a screening programme staff need to be motivated to participate. Within this review staff motivation was identified as a key determinant of screening success. This concept is supported in the wider literature where opportunities to improve patient care and patient outcomes are identified as clear motivators for staff to engage in screening and appropriate referral in the ED (Liu et al., 2022 [20]; Hawk and D’Onofrio, 2018 [13]; Eagles, 2022 [10]). Similarly, this synthesis highlights that commitment to the patient, maintaining patient safety and adequately responding to those who required screening e.g. post fall, were significant motivators to engage in screening. Furthermore, rewards and incentives in the form of positive feedback on screening practices was a clear motivator for staff, however, staff were mainly concerned with how this impacted the patient and resulted in positive outcomes and a consequential positive effect on them personally. Therefore, emphasising and reinforcing the importance and impact of screening in the ED cannot be underestimated by those seeking to implement screening and engage staff successfully in the process. Empowering staff in the ED has been linked to a number of variables including environment, successful collaboration, work effectiveness, autonomy and job satisfaction (Devivo et al. 2013 [8]; Tavender et al., 2014 [31]). Similarly, this synthesis identified motivation factors including ensuring multidisciplinary collaboration and adequate organisational supports through education, leadership and mentorship in the ED. A supported workforce felt empowered to screen and maintained screening practices. The stressful and unpredictable ED environment is a clear barrier with local and organisational culture impacting on successful implementation of screening (Liu et al., 2022 [20]; Eagles, 2022 [10]). This synthesis further highlights that competing interests in the ED, environmental stressors such as workload, overcrowding and time pressures and an organisational culture that resists screening and does not adequately support ED staff were clear barriers to attaining motivated screening practitioners.

### Knowledge and skills to screen

The importance of knowledge and skills development to ensure accurate and consistent screening is well recognised (Harley et al., 2019 [12]; Tavender et al., 2014 [31]; Liu et al., 2022 [20]). However, to give further insight into ED staffs behaviour, this synthesis ascertained that ED staff consciously develop skills and acquire knowledge to respond to patients who required screening and competently engage in screening practices. Similar to Tavender et al. (2014) [31], access to educational resources and staff supports was deemed vital to ensure successful implementation. Orientating staff to the process through tailored education and workshops resulted in knowledgeable screening practitioners and reinforcing the screening process by increasing awareness and providing adequate and updated information was a further facilitator. Theoretical and practice-based skills training, tailored to the site and patient cohort need to be readily available for staff and this is also consistent with the current evidence base (Tavender et al., 2014 [31]; Liu et al., 2022 [20]). Not surprisingly, specific barriers to skills development included ED staff turnover, high work volume or the need to attend to critically ill patients where resources were often directed away from the recognition and response to patients who required screening and referral.

Resource limitations, a lack of culture change and cumbersome bureaucratic structures are well recognised as barriers to implementing change and understanding local policy creation in the ED (Shaikh et al., 2018 [30]). This synthesis further identified screening specific barriers to knowledge and skills development with staff finding screening protocols difficult to recall, particularly in a busy ED environment where constant updates complicated the process and were difficult to adhere too. Complex screening processes and a lack of procedural knowledge also created barriers for staff to overcome. However, healthcare workers were keenly aware of their own knowledge deficits around screening but often are unsure of how to address these deficits and required experienced staff and education to assist them. HCWs described an unwillingness and discomfort in engagement in screening if they did not receive adequate support or training and some felt they lacked the skills and competence to engage in complex forms of screening where patients had co-morbidities and complicated histories. Clarification of each individuals’ responsibility pertaining to screening and assessment is vital to instil professional confidence and improve detection and management, (Kennelly et al., 2013 [15]; Crilly et al., 2020 [7]).

Successful implementation requires monitoring of new departmental policies, required infrastructure and teamwork (Shaikh et al., 2018 [30]). Similarly, this synthesis found that ready access to resources that would underpin screening were considered vital, particularly with what was considered to be more complex forms of screening, sepsis being a prime example. Furthermore, access to memory aids such as algorithms and simple protocols developed with staff and monitoring adherence of new screening practices through staff assessment helped to resolve these knowledge deficits. Similar to Shaikh et al. (2018) [30], a lack of willingness to change practices within the ED was also a barrier with a lack of time to update knowledge and skills described as a consistent challenge. 

### Study limitations and strengths

There are several strengths of this Qualitative Evidence Synthesis. We have provided new insights in terms of the barriers and facilitators to screening in the ED. The review was conducted using established systematic processes to ensure rigour and quality were maintained throughout, and bias minimised in terms of literature searching, screening, appraisal, and synthesis. Furthermore, to ensure reflexivity, authors discussed, examined and considered the significance of their beliefs, attitudes and perceptions surrounding the question and methodology during each stage of synthesis (Larkin et al., 2019 [18]; Barry et al., 2021 [3]). The resulting high agreement between researchers enables confidence in the results reported. As reported, we had moderate- high confidence in the review findings using GRADE CERQual assessment criteria, indicating studies were conducted in a rigorous manner. In terms of transferability of findings, the number of screening modalities explored [11] and the findings linked with the ED (23 studies from ED settings) are a strength of the review. However, geographical spread was limited to mainly Australia, UK or North America highlighting the need for further research in this area internationally. Screening is also taking place more widely in acute assessment units, injury units and specialist acute settings. These settings were included to a limited extent (SAU, MAU) and would require greater exploration to ensure rigour when applying findings to these sites. This would ensure context specific recommendations. Predominantly, nurses and doctors were interviewed and surveyed to inform the studies included in this review. However, the evidence would suggest that screening is a multifaceted process involving multiple members of the MDT team at different stages of the patient pathway through the ED. Therefore, this is a limitation of the review as available evidence included little from MDT perspective outside of nurses and doctors.

### Clinical and policy implications

Ensuring that screening is part of the ED culture and that staff are educated and supported in engaging in screening is vital. Furthermore, an in-depth understanding of local ED culture and attitudes towards screening must be attained to ensure successful implementation. Current screening practices in each ED/Assessment Unit must also be reviewed and audited to establish a baseline for implementation and required infrastructure.

The successful implementation of screening is also dependent on managerial support, leadership and the establishment of educational, practice development, and support structures to underpin the process. These QES findings can be of value currently to inform guideline, policy or protocol development or assist those attempting to implement screening in acute care settings. Findings pertain to multiple forms of screening and therefore are adaptable and can be tailored to inform a plethora of screening modalities.

### Areas for further research

As reflected in the QES findings, screening is used by multiple healthcare practitioners. A qualitative study which explores the experience of the wider MDT whilst screening in the ED would provide greater insight into relevant barriers and facilitators. These findings may be more contextually relevant and underpin the screening process. In addition, assessing the educational needs of ED staff and current screening practices at local level is vital to inform tailored implementation processes. Therefore, consultation with those who are involved or potentially involved in the process is vital, particularly at the beginning of the process. This could also be achieved by interviewing or consulting with relevant staff and consequently gaining an in-depth understanding of local ED culture and behaviours.

Nationally, screening practices and the patient pathway through the ED vary greatly between sites, consequently, it is vital to attain local insight into relevant barriers and facilitators. There is a distinct lack of Irish research to inform local implementation processes. In addition, consulting other relevant stakeholders including patients, family members and the wider interdisciplinary team both in the community and primary care services would give greater insight into possible expansion of implementation and application of screening to identify patients at risk of adverse outcomes. Research in this area must ultimately inform the development and implementation of evidence-based care pathways following screening to support patient care.

## Conclusion

This paper offers a context specific findings to explain and describe the barriers and facilitators to screening in the ED. This knowledge adds to the evidence base that informs implementation strategies and planning around screening. However, more can be learned by consulting those who are at the frontline of acute services to inform successful implementation strategies.

Healthcare workers are motivated to engage in screening if involved in the implementation process and offered education and support to sustain screening practices in an often-challenging environment. Competing interests pertaining to the care of acutely ill patients impacts adherence to screening. Furthermore, those engaged in screening must have in-depth knowledge of the process and an understanding of the benefits for the patient, the ED environment and their professional practice to ensure sustained adherence.

### Supplementary Information

**Additional file 1: Supplementary file 1.** Medline Search String.

**Additional file 2: Supplementary file 2.** Rationale for exclusion of full text articles.

**Additional file 3: Supplementary file 3.** CASP critical appraisal of included studies.

**Additional file 4: Supplementary file 4.** Descriptive characteristics of included studies.

**Additional file 5: Supplementary File 5.** Theoretical Domains Framework Atkins 2017 Definitions and Constructs Table.

**Additional file 6: Supplementary file 6.** Sample of Coding Excerpts from Article No 13 Menser et al (2015) Organised under Domains with Related Constructs.

**Additional file 7: Supplementary file 7.** Findings Categorised under TDF Domains.

**Additional file 8: Supplementary file 8.** Mapping of Barriers and Facilitators to Final Themes.

**Additional file 9: Supplementary file 9.** GRADE CERQual Confidence in Findings.

## Data Availability

The datasets used and/or analysed during the current study available from the corresponding author on reasonable request.
