# Supplementary material for: A qualitative evidence synthesis (QES) exploring the barriers and facilitators to screening in emergency departments using the theoretical domains framework
Source: BMC Health Serv Res. 2023 Oct 11;23:1090. doi: 10.1186/s12913-023-10027-3 (PMC10568862; doi:10.1186/s12913-023-10027-3)
Supplement: Supplementary file 1 — Additional file 1: Supplementary file 1. Medline Search String. [file 12913_2023_10027_MOESM1_ESM.docx]

**Supplementary file 1: Medline Search String**

|  | Barriers*(barriers or obstacles or challenges or difficulties or issues) OR Factors* (factors or causes or influences or reasons or determinants or predictors) OR Facilitators* (facilitators or motivators or enablers) OR Barriers OR facilitators* |
| --- | --- |
| **AND** | Screenin* OR Screening Too* OR Screening Instru* OR Screening Mea*  Assessment* OR Assessment Tools OR Assessment method OR Assessment Strategy OR Clinical Assessment OR Clinical Assessment Tools* |
| **AND** | Emergency department* OR Emergency room OR Emergency Ser* OR Emergency Care* OR Emergency Medi* Accident and emergency OR Accident & emergency OR a&e OR a & e |
|  | qualitative OR experience* OR perception* OR perspective* OR case stud* OR interview* OR focus group* OR mixed methods OR participant observation OR transcript* OR ethnograph* OR phenomenol* OR grounded theor* OR grounded-theor* OR purposive sample OR lived experience* OR narrative* OR life experience* OR life stor* OR action research OR observational method OR thematic analysis OR narrative analysis OR field stud* OR field-notes OR video-recording |
