# Supplementary material for: A qualitative evidence synthesis (QES) exploring the barriers and facilitators to screening in emergency departments using the theoretical domains framework
Source: BMC Health Serv Res. 2023 Oct 11;23:1090. doi: 10.1186/s12913-023-10027-3 (PMC10568862; doi:10.1186/s12913-023-10027-3)
Supplement: Supplementary file 2 — Additional file 2: Supplementary file 2. Rationale for exclusion of full text articles. [file 12913_2023_10027_MOESM2_ESM.docx]

**Supplementary file 2: Rationale for exclusion of full text articles**

| **Article Full-Text Review** | **Category** | **Rationale for Exclusion** |  |
| --- | --- | --- | --- |
| Arslanian-Engoren, (2018) | Wrong Focus | Sole focus on Triage within the Emergency Department. |  |
| Barmentloo et al (2020) | Wrong Design | Did not use qualitative methods for data collection and analysis of data.  Did not include the experience of stakeholders who are involved in the screening process. |  |
|  |  |  |  |
| Bendtsen et al (2007) | Wrong Design | Did not use qualitative methods for data collection and analysis of data. |  |
| Boult et al (1998) | Relevance | Wrong publication type. Opinion piece. |  |
| Braxter et al (2014) | Wrong Population | Did not include the experience of stakeholders who are involved in the screening process. |  |
| Brouwers et al (2017) | Wrong Focus | Focus on provision of care rather than screening. |  |
| Byhoff et al (2018) | Wrong Population | Parents/carers of paediatric patients. |  |
| Carpenter et al (2016) | Relevance | Wrong publication type. Discussion paper. |  |
| Freiermuth et al (2015) | Wrong Population | Did not include the experience of stakeholders who are involved in the screening process. |  |
| Gesell et al (2018) | Wrong Focus | Focus on implementation of a care pathway. No clear focus on screening. |  |
| Hayes et al (2019) | Wrong Focus | Focus on patient transfers. No clear focus on screening. |  |
| Heinert et al (2017) | Wrong Focus | Focus on HIV screening programs. No clear focus on screening for functional decline. |  |
| Jeffs et al (2012) | Relevance | Focus on care evaluations and guidance, no clear guidance on screening. |  |
| Jelinek et al (2013) | Wrong Population | Focus on screening of adults with mental health presentations. |  |
| Johansen et al (2011) | Wrong Focus | Sole focus on triage within the Emergency Department. |  |
| Kappen et al (2016) | Relevance | Focus on prediction models in practice. No clear focus on screening for functional decline |  |
|  |  | or adverse outcomes. |  |
|  |  |  |  |
| Kerber et al (2017) | Wrong Focus | Limited focus on screening, main focus on a single tool and not on barriers and facilitators to the screening process. |  |
|  |  |  |  |
| Lam et al (2016) | Relevance | Focus on guideline usage. No clear focus on screening for functional decline or adverse outcomes. |  |
| Leblanc et al (2012) | Wrong Focus | Focus on HIV screening. Full text not available in English. |  |
| MacWilliams et al (2017) | Wrong Focus | Focus on screening of paediatric patients. |  |
| Mahabee-Gittens et al (2014) | Wrong Focus | Focus on screening in the Paediatric ED. |  |
| Meurer et al (2011) | Relevance | Focus on guideline implementation and not screening. |  |
| Mondragón et al (2008) | Wrong Focus | Focus on alcohol consumption. |  |
| Mulindwa and Blitz (2016) | Wrong Focus | Sole focus on triage within the Emergency Department. |  |
| Mullen et al (2018) | Wrong Focus | Focus on a Paediatric screening tool. |  |
| Murphy et al (2019) | Relevance | Focus on intervention strategies and not on screening tools or the screening process. |  |
| Karlsson et al (2005) | Wrong Focus | Focus on alcohol dependence screening. No clear focus on screening for functional decline or adverse outcomes. |  |
| Nordqvist et al (2006) | Wrong Focus | Alcohol screening and intervention is the main focus. |  |
| O’Malley et al (2013) | Relevance | Article based on paediatric/family assessment. |  |
| Pailler et al (2009) | Wrong Population | Focus on mental health-based screening. |  |
| Sormanti and Smith (2009) | Wrong Focus | Focus on intimate partner violence screening. |  |
| Sorsdahl et al (2014) | Wrong Focus | Focus on substance misuse. |  |
| Tanabe et al (2010) | Wrong Focus | Sole focus on sickle cell disease, outcomes do not pertain to screening for functional decline or adverse outcomes. |  |
|  |  |  |  |
| Damme et al (2020) | Wrong Setting | Wrong setting. Not related to the Emergency Department. |  |
| Warburton et al (2004) | Relevance | Currency of evidence to inform the QES. Does not meet inclusion criteria. |  |
| Van der Westhuizen et al (2019) | Wrong Focus | Focus on substance misuse. |  |
| Watkins et al (2019) | Wrong Outcome | No clear outcome that pertains to screening within the Emergency Department. |  |
| White et al (2016) | Wrong Focus | Focus on HIV and Hepatitis C Screening Program. |  |
| Wiskel et al (2019) | Wrong Focus | Focus on HIV and Hepatitis C Screening Program. |  |
| Wolf et al (2018) | Wrong Focus | Sole focus on triage within the Emergency Department. |  |
| Wallace et al (2021) | Wrong Focus | Focus on social needs screening/Social determinants |  |
| Bray and Kennedy (2021) | Wrong Outcome | Focus on sepsis care as opposed to screening |  |
| Billah et al (2022) | Wrong Outcome | Not pertaining to Screening. |  |
| Wallace et al (2020) | Wrong Focus | Focus on social needs screening/Social determinants |  |
