# Supplementary material for: A qualitative evidence synthesis (QES) exploring the barriers and facilitators to screening in emergency departments using the theoretical domains framework
Source: BMC Health Serv Res. 2023 Oct 11;23:1090. doi: 10.1186/s12913-023-10027-3 (PMC10568862; doi:10.1186/s12913-023-10027-3)
Supplement: Supplementary file 3 — Additional file 3: Supplementary file 3. CASP critical appraisal of included studies. [file 12913_2023_10027_MOESM3_ESM.docx]

**Supplementary file 3: CASP critical appraisal of included studies**

| Study ID | Context described | Sampling strategy appropriate and described | Data collection strategy appropriate and described | Data analysis appropriate and described | Findings supported by evidence | Evidence of researcher reflexivity | Ethical issues been taken into consideration | Any other concerns | Overall assessment of methodological limitations | Quality Rating |
| --- | --- | --- | --- | --- | --- | --- | --- | --- | --- | --- |
| Abdulwahid et al (2018) | Yes | Yes | Yes | Yes | Yes | Yes | Yes | Yes, Further Detail on Methodology | Minor | Mod |
| Crilly et al (2020) | Yes | Yes | Yes | Yes | Yes | Yes | Yes | No | Minor | Mod |
| Daniel et al (2015) | Yes | Yes | Yes | Yes | Yes | Yes | Yes | No | Minor | Mod |
| Fry et al (2016) (A) | Yes | Yes | Yes | Unclear | Yes | Unclear | Yes | No | Moderate | Mod |
| Fry et al (2016) (B) | Yes | Yes | Yes | Yes | Yes | Yes | Yes | No | None | High |
| Gorawara-Bhat et al (2017) | Yes | Yes | Yes | Yes | Yes | Yes | Yes | Yes, Further Detail Ethical Considerations | Minor | Mod |
| Gwyther et al (2018) | Yes | Yes | Unclear | Unclear | Yes | Unclear | Unclear | Yes, Methodology difficult to ascertain fully, not ED origin | Major | Low |
| Hoyle and Grant (2015) | Yes | Yes | Yes | Yes | Yes | Unclear | Yes | Yes, clarification of possible bias. | Minor | Mod |
| Harley et al (2019) | Yes | Yes | Yes | Yes | Yes | Yes | Yes | No | None | High |
| Kirk and Nilsen (2015) | Yes | Yes | Yes | Yes | Yes | Yes | Yes | No | None | High |
| Kirk et al (2016) | Yes | Yes | Yes | Yes | Yes | Yes | Yes | No | None | High |
| McEwan et al (2018) | Yes | Yes | Yes | Yes | Yes | Unclear | Yes | Further information on the role of the researcher | Minor | Mod |
| Menser et al (2015) | Yes | Yes | Yes | Yes | Yes | Yes | Yes | No | None | High |
| Midori-Sakai et al (2016) | Unclear | No | No | No | Unclear | No | No | Questionable credibility of source, poor translation. Inappropriate presentation of methodology, very unclear evidence underpinning methods etc. | Major | Low |
| Mistry et al (2018) | Yes | Yes | Yes | Yes | Yes | Yes | Yes | Further information pertaining to ethics required | Minor | Mod |
| Munroe et al (2017) | Yes | Yes | Yes | Yes | Yes | Yes | Yes | No | None | High |
| O'Keeffe McCarthy et al (2014) | Yes | Yes | Yes | Yes | Yes | Yes | Yes | Yes, Further Detail Ethical Considerations | Minor | Mod |
| Olson et al (2011) | Yes | Yes | Yes | Yes | Yes | Yes | Unclear | Some ethical considerations evident but permissions by ethical review board. | Minor | Mod |
| Pirotte et al (2014) | Yes | Yes | Yes | Yes | Yes | Yes | Yes | No | None | High |
| Puchalski-Ritchie et al (2019) | Yes | Yes | Yes | Yes | Yes | Yes | Yes | No | None | High |
| Roberts et al (2017) | Yes | Yes | Yes | Yes | Yes | Yes | Yes | No | None | High |
| Salkeld et al (2011) | Yes | Yes | Yes | Yes | Yes | Yes | Yes | No | None | High |
| Sampson et al (2019) | Yes | Yes | Yes | Yes | Yes | Yes | Yes | No | None | High |
| Schoenfield et al (2019) | Yes | Yes | Yes | Yes | Yes | Unclear | Unclear | Possible bias between researchers and participants. | Minor-Mod | Mod |
| Skyttberg et al (2016) | Yes | Yes | Yes | Yes | Yes | Yes | Yes | No | None | High |
| Tarrant et al (2016) | Yes | Yes | Yes | Yes | Yes | Yes | Yes | No | None | High |
| Tavender et al (2014) | Yes | Yes | Yes | Yes | Yes | Yes | Yes | No | None | High |
| Van der Wulp et al (2011) | Yes | Yes | Yes | Unclear | Yes | Unclear | Unclear | Yes, methodology difficult to ascertain fully with regards to analysis, possible bias and | Moderate | Mod |
| Eagles (2022) | Yes | Yes | Yes | Yes | Yes | Yes | Yes | No | None | High |
| Wolf et al (2019) | Yes | Yes | Yes | Yes | Yes | Yes | Yes | No | None | High |
