# Supplementary material for: A qualitative evidence synthesis (QES) exploring the barriers and facilitators to screening in emergency departments using the theoretical domains framework
Source: BMC Health Serv Res. 2023 Oct 11;23:1090. doi: 10.1186/s12913-023-10027-3 (PMC10568862; doi:10.1186/s12913-023-10027-3)
Supplement: Supplementary file 4 — Additional file 4: Supplementary file 4. Descriptive characteristics of included studies. [file 12913_2023_10027_MOESM4_ESM.docx]

**Supplementary file 4: Descriptive characteristics of included studies**

| Author, Yr, Country | Data Collection | Method of Analysis | Study Aims and Objectives | Sample | Participant Characteristics | Relevant Findings for Present Study | Study Quality |
| --- | --- | --- | --- | --- | --- | --- | --- |
| Abdulwahid et al, (2018) UK  Article No 1 | Semi-structured Telephone Interviews | Template Analysis. NVIVO 11 for Final Analysis | To gain an understanding of Senior Doctor Triage (SDT) from the perspective of emergency hospital staff. This was the first National study within the UK to explore the experiences/opinions of various emergency and managerial staff on the SDT model which was inclusive of staff and organisational dynamics, screening and assessment and implementation methods. | 27 Participants Convenience Sampling of 13 ED Consultants then Snowballing Sample of 4 nurses, 7 paramedics, 2 ED managers and 1 junior Doctor. | The senior doctors interviewed were consultants with experience ranging from 3 to 10 years; all of the nurses were registered nurses with experience ranging from 3 to 20 years while the paramedics’ years of clinical experience ranged from 6 months to 20 years. | This study identified barriers and facilitators of the SDT process and analysed ED staffs views of this model of care. Relevance pertains to the implementation of triage, screening and assessment within the ED. Knowledge of the perceived positive and negative aspects, enablers and facilitators was included. . | Moderate |
| Crilly et al, (2020) Australia  Article No 2 | One on one Semi-structured Interviews | Coding Using Theoretical Domains Framework (TDF), Content Analysis to Identify Domains, Thematic Analysis to Identify Themes | The aim of this study was to use the TDF to examine clinicians’ use of the Improved Assessment of Chest Pain Trial (IMPACT) protocol, whether there were any barriers or enablers to protocol use, and whether there are further opportunities to implement IMPACT. | 9 participants. Medical (n = 4) Nursing (n = 5) Staff working at the Emergency and Trauma Centre where the protocol was implemented. Purposive Sampling. | Participants included staff (doctors and nurses) employed on a full-time or part-time basis. Three participants were male and the average age of participants was 40 years (SD: 10.3). The average years of experience in their profession was 17 (SD: 9.2), with 12.6 years (SD: 6.6) working in the ED. | Risk stratification and management of patients with Acute Coronary Syndrome and description of the barriers and facilitators to the implementation of this protocol in the ED setting.  ED staff identified domains that influenced their chest pain management behaviour. Enabled and supported protocol use and champions/leaders that were trusted and accessible were facilitators. Further implementation opportunities were identified. | Moderate |
| Daniel et al (2015) Australia  Article No 3 | Observation of Triage Nurses/ Semi-structured Interviews, Field Notes. | Thematic Analysis. | To explore the need for a violence risk screening process in an Australian ED. To assess the feasibility of the screening process at triage by observing how triage nurses screen for risk for violence (Screening Tool). | Triage nurses (N=9) were observed conducting triage assessments (N=167) over 30 hours. Convenience Sampling. | Triage nurses with experience of violence risk screening were observed. Self-nominated to participate and offered an in-service education session. Duration of observation 4hrs/nurse | Observation revealed that the existing screening approach was not being consistently used. The relevance of findings pertain to the implementation, infrastructure and staff development/training needs and attitudes, not specific to violence screening alone. | Moderate |
| Fry et al (2016) Australia (A)  Article No 4 | Focus Groups | Thematic Analysis | The aim of the study was to understand emergency nurses’ perceptions of the management of acute pain for older persons with cognitive impairment and presenting with a long bone fracture. | 80 ED emergency nurses participated. Purposive Sampling. | 67 (84%) females and 13 (16%) males, in 16 focus groups across four emergency departments. Nurses had an average of 12.5 years as a Registered Nurse (SD ± 10.06) and 8.6 years (SD ± 8.64) emergency experience. Participants had to have at least one year of experience in the ED | The lack of a standardised pain assessment screening tool in the ED was a significant barrier identified by ED staff. Belief in championing and leadership in this area was deemed vital. In-depth insights into the barriers and facilitators of practice assessment and the nurses’ role in relation to same within the ED were described that apply to screening and screening processes. | Moderate |
| Fry et al (2016)  Australia (B)  Article No 5 | Semi-structured Focus Groups | Thematic Analysis  Constructivist Theory to interpret meaning and NVIVO Coding | To explore emergency nurses’ perceptions of the feasibility and utility of Pain Assessment in Advanced Dementia tool in people over 65 with cognitive impairment. The objective was to determine which observational pain assessment tool was the most appropriate for the emergency department context and the cognitively impaired older person. | 6 Focus Groups with 36 nurses across three hospital EDs. Purposive Sampling | ED nurses had to have a minimum of a bachelor’s degree or an equivalent who had used the PAINAD tool within their practice. | Participants found the PAINAD tool useful but also valued the input of family and carers in the process and to inform the assessment and management of pain in this group. This study offers ED nurses perspective on the detection, assessment and management of pain among cognitively impaired individuals in the ED. This was viewed as a challenging, complex process. | High |
| Gorawara-Bhat et al (2017)  USA  Article No 6 | One on one, face to face interviews | Thematic Analysis | To understand, in their own words, ED nurses’ perceptions of assessing older-patients’ pain and use emergent themes to guide optimal interventions for improving the quality of pain assessment and management in the ED. | 20 ED Nurses working in an adult urban ED. Convenience sample with varying academic background, experience and ED specific experience. | 30% of nurses employed were interviewed. A diverse sample group  3: Associates Degree, 15: BSc, 2: MSc, 1: ER Tech.  Experience (4) <5 yrs (4) 5-10 yrs  (7)11-20 yrs (5) >20 yrs. (13) <5 years Exp in the ED, (6) had 5-10 years Exp, (1) had 11-20 years Exp. | Typology framework to guide the development of pain assessment tools and pain management. New combinations are needed for assessing multi-dimensional pain in older-patients. These findings give insight into the implementation of these tools in the ED. | Moderate |
| Gwyther et al (2018)  Europe  Article No 7 | Online Survey with Open Responses | Thematic Analysis | This study explores the experiences of partners delivering frailty interventions within Europe, registering their programmes with the European Innovation Partnership on Active and Healthy Ageing (EIP-AHA). | 21 partners in 7 countries delivering frailty interventions.  Purposive Sampling. | Partners who had implemented frailty programmes were recruited due to their experience. Five partners stated that their current activities did not match the survey brief and were removed from the sample. | More in-depth responses were required and illustrated that there was a lack of consistency between EIP-AHA partners in methods of defining, screening and measuring for frailty and pre-frailty. An increased proportion of interventions that consistently apply valid methods of screening and/or measuring frailty and pre-frailty are required. Working with Stakeholders and Project Management were the themes identified under which barriers and facilitators were described. | Low |
| Hoyle and Grant (2015)  UK  Article No 8 | Semi-structured Interviews. | Thematic Analysis | To understand nurses’ views and experiences of four hour treatment targets in the ED and how this impacts clinical decision-making throughout acute secondary care hospitals. | 31 Nurses working in UK Secondary Care Hospital which had an ED. | Purposive Sample  (6) M (25) F.  Emergency Arenas:  ED (5), Medical Assessment (4), Surgical Receiving (2)  Surgical Wards: 11  Medical Wards: 9  Qualified: (1) 1-2 yrs  (11) 3-5 years, (6) 6-10 years, (13) > 20 years. Band 5 (Staff Nurse Level) (20) Band 6 (4) Band 7 (Manager) (7) | These targets were not viewed as helpful or appropriate by staff. Barriers and facilitators to the implementation of new practices, processes in these settings were described. Relevance: Factors which may impact on decision-making and practitioner driven allocation and screening of patients were described. | Moderate |
| Harley et al (2019) Australia  Article No 9 | Semi-structured Interviews. Face to face. | Thematic and Consensus-based Content Analysis | This study explored ED nurses’ experiences and perceptions around recognising and responding to patients with sepsis, and their awareness of sepsis screening and prognostic tools. | 14 Registered Nurses (RNs) Employed in the ED. Purposive Sample. | Registered Nurses (RNs) who worked clinically in the ED who had previously cared for a patient with sepsis. | Environmental and organisational barriers and the importance of experiential learning and valuable methods of educating staff were identified as barriers and facilitators to nurses’ knowledge development and understanding of their role in sepsis management. Deficits in nurses’ capacity to recognise and respond to patients with sepsis were identified. | High |
| Kirk and Nilsen (2015) Denmark  Article No 10 | Semi-structured, Face to face Interviews. Ethnographic fieldwork (3 months). | Contradiction Analysis, Activity System Analysis, Cultural Historical Activity Theory. | How does the flow culture in an emergency department influence nurses’ use of a research-based clinical guideline and a nutrition screening routine? | Nurses, medical secretaries and doctors in the emergency department were followed/observed. | Nurses routinely performed screening in the ED for nutrition risk to identify those who require nutritional support. They were observed engaging in this process. | We found that research-supported guidelines and screening routines were not used if they were perceived to stop the patient flow, suggesting that the practice was not fully evidence based. These ED findings provide insight into the barriers and facilitators to the implementation of guidance and nutirional screening in the ED. | High |
| Kirk et al (2016) Denmark  Article No 11 | Focus Group Interviews,  Semi-structured Interviews | Theoretical Domains Framework with Content Analysis whereby new themes and themes already under each domain were described. | The aim was to identify factors that were perceived as most important as facilitators or barriers to the introduction and intended use of a new screening tool in the ED among nurses and a geriatric team. This tool identified patients at higher risk of readmission or functional decline. | 8 ED Nurses (7) F (1) M. Participated in 2 Focus Groups, 4 Each Group.  Geriatric team (1 nurse, 2 physiotherapists, a secretary and on consultant) one focus group. | 5 managers, nursing and geriatric team were interviewed individually so their position would not impact on the responses in the focus groups. Mixed or age | Different ED cultures can exist in the same local context and can influence perception of barriers and facilitators differently. These cultures must be identified and addressed if implementation of a new screening tool is planned. Different professional roles and responsibilities and different actions and sense-making impacted on how screening tools were perceived in the ED. | High |
| McEwan et al (2018)  UK  Article No 12 | Direct Observation of ED Staff. Review of Clinical Records of Interactions. Semi-structured interviews with healthcare professionals. | Framework analysis assisted utilising NVIVO. | The aim was to ascertain how falls are managed in ED's. The rationale for the lack of adherence to guideline recommendations in the ED was also ascertained. Exploration of the barriers and facilitators influencing adherence to guidelines in the ED's and the identification of the ways barriers could be addressed. | The study involved two EDs. 27 episodes of observation of healthcare professional interactions with patients aged 65 or over presenting with a fall, supported by review of the clinical records of these interactions, and subsequently, 30 interviews with healthcare professionals. | 30 professionals (20 doctors, 10 nursing staff) were observed delivering 27 episodes of care. Of the 27 patients, 19 were male and 8 female, ranging in age from 67 to 98. Doctors (20) and nursing staff (10) were interviewed post observation and chart review. Sampling was opportunistic. | Support from senior staff; education; cross-boundary care; definition of falls; communication; organisational factors and staffing all influenced adherence to guidelines in the ED. Simple interventions such as education and pro-formas are unlikely to have substantial effects alone. However, taking advantage of the influence of senior staff on juniors could enhance adherence. | Moderate |
| Menser et al (2015)  Article No 13 | Primary data collection including document review, internal stakeholder interviews, and direct observation of program processes were used for this formative evaluation of program implementation. One on one interviews with staff. | Data analysis using a constant comparative approach. | The aim of this study was to use formative evaluation to describe program goals, process, and early implementation experiences at 2 ED sites that adopted a medical screening program before wider implementation within a rural health system. | Purposive sample  Fourteen key informants were asked questions related to the program concept, structure, and implementation at 2 sites. | All respondents were clinical or administrative employees or affiliates of the health care system. Age, Gender, Years of experience and educational level of participants was also detailed. | Relevant as it discusses implementation processes, barriers and facilitators and knowledge relevant to the implementation of medical screening in the ED. The program, as implemented, aligned with initial program goals, but it was dependent on ED screening staff and rural healthcare availability. | High |
| Midori Sakai et al (2016)  Brazil  Article No 14. | One on one semi-structured interviews with ED Nurses | Content Analysis: Pre analysis, Analytical Description and Interpretation Framework. | The aim of this study was to explore the feelings of nurses who undertake risk assessment and classification in the ED of a public hospital. | Purposive Sample of 12 ED Nurses who engaged in risk assessment and classification in the ED. | ED Nurses who Risk Assess Patients. No other demographic data provided on participants. | Barriers and facilitators to undertaking risk assessment and classification in the ED were identified. This includes increased autonomy to advocate for patients and barriers pertaining to a lack of resources. | Low. |
| Mistry et al (2018)  USA and UAE  Article No 15 | One on one, semi-structured interviews of emergency triage nurses. | Content Analysis using NVivo. | To explore emergency nurses perceptions of the Emergency Severity Index (ESI) to identify strengths, weaknesses and barriers to implementation internationally. | Purposive sample of triage nurses who volunteered to be interviewed. Each nurse had undergone structured training in the use of the ESI using standardised online modules and competency assessment. | 27 Nurses were interviewed who worked in the ED of a 165 bedded community hospital. Age, Gender, Years of Experience as a Nurse using ESI and Triage based systems was also collected. | Barriers and facilitators to the use of the ESI were identified. Subjective of score assignment, level of nurse experience in using the ESI and subjectivity in pain experience were identified as issues. | Moderate |
| Munroe et al (2018)  Australia  Article No 16 | Focus groups at the end of the study day, to gain further insight into participant beliefs. | Thematic Analysis using NVivo. | To determine potential facilitators and barriers and tailor interventions to optimise future implementation of a patient-assessment framework into emergency nursing practice. | 38 early career emergency nurses from 5Australian hospitals participated in an education workshop on HIRAID assessment framework. All participants completed surveys,interviews and focus groups to identify potential facilitators and barriers. 23 participants completed follow-up telephone surveys 4–6 months later. | Registered Nurses with<3 years post-registration experience currently working in an emergency department were selected to participate. Nurse Managers, Educators and Clinical Nurse Consultants from study sites reviewed the post graduate experience of nursing staff employed in the ED and 86 eligible nurses from the six hospital sites were identified to meet the inclusion criteria | To optimise future implementation, educational workshops, environmental restructuring, modelling and social supports are required. A multimodal strategy is needed to promote future successful implementation of the HIRAID assessment framework into emergency nursing practice. | High |
| O'Keeffe McCarthy et al (2014)  Article No 17 | Focus Groups |  | The aim was to gain the perspectives of ACS patients and emergency staff nurses on the rural patient experience of cardiac pain and anxiety and priorities and barriers to optimal assessment and management of ACS pain. |  |  |  | Moderate |
| Olson et al (2011)  USA  Article No 18 | One on one semi-structured interviews with stroke personnel who practice out of the ED. | Thematic Analysis | The purpose of this study was to provide an understanding of the professional opinions of the staff in hospitals that have achieved a high percentage of success with timely IV Tpa administration. | Purposive sample of key stroke personnel across seven top performing sites contributing data to the GWTG-Stroke registry. | 13 staff were interviewed. These included stroke coordinator/managers, Neurologist, Radiologist, Nurse Manager, Pharmacist and Physician. | Barriers and facilitators to shorter door-needle time were identified. Key areas identified were communication and teamwork, Organisational culture, performance monitoring and feedback and overcoming barriers. | Moderate |
| Pirotte et al (2014)  USA  Article No 19 | One on one semi-structured interviews. | Content Analysis | The aim of the study were to determine characteristics of an intervention that would persuade patients to follow-up with a primary care physician for further BP evaluation and encourage clinicians to screen for BP. | Study conducted at a large, urban academic ED. Stratified Sampling where clinicians were approached to be interviewed. | 9 ED Physicians and 8 ED Nurses were interviewed. | To successfully screening for hypertension, the SBIRT intervention must be simple, easy to implement and include automated processes to orientate staff. Additional barriers and facilitators to screening were also identified. | High |
| Puchalski Ritchie et al (2019)  Canada  Ethiopia  Article No 20 | Semi-structured interviews with clinical, administrative and support services staff. | Direct Content Analysis using NVivo. | Aim was to identify barriers and facilitators to utilization of EBCAs Evidence-based clinical algorithms in the TASH-ED Tikur Anbessa Specialized Hospital emergency department (TASH-ED), to identify priority targets for development of EBCAs tailored for the TASH-ED context and to understand the process of care in the TASH-ED to inform implementation planning. | Purposive sampling and subsequent snow balling and a rigorous recruitment strategy with inclusion and exclusion criteria and an organised collaborative process developed in conjunction with the sites. | 26 key informants were interviewed. 18 TASH staff and 8 University of Toronoto. This included ED Consultants, Nursing and Administrative Staff and ED residents. Level of experience, training were detailed. Direct observations of staff during this process also informed the data collection. | Barriers and facilitators were clearly identified to the implementation of EBCAs. Lack of medication, equipment and human resources are identified as primary barriers. Support from leadership and engagement of stakeholder from outside the ED were recognised as facilitators. Direct observations, interviews and analysis of documentation were proposed to answer the research study and address the outlined objectives. | High |
| Roberts et al (2017)  UK  Article No 21 | One on one semi-structured interviews with staff in an acute hospital ED in 760-bed district general hospital | Content Analysis using a coding framework to promote greater coding consistency. | The aim of this study is to use a behavioural science approach to identify barriers and facilitators towards Sepsis Six implementation in a case study hospital. | Purposive sample of relevant stakeholders, Nurses, Junior and Consultant Level Doctors, working in the ED, SAU and MAU. Stratified sample of 3 participants at the start of the process to refine methods. | Overall, approx. 16 participants were interviewed about their experience in implementing the sepsis six bundle. Participant demographics were detailed and a diverse cohort were interviewed to inform the study. | Important barriers were identified including poor feedback and communication, insufficient training and lack of resources to support the sepsis six bundle implementation. Facilitators include knowledge and skills to perform screening and beliefs in the benefits of the bundle. | High |
| Salkeld et al (2011)  Canada  Article No 22 | Small Focus Group Interviews with ED staff involved in the program | Framework Analysis Approach. | To describe the general characteristics of an ED based clinical decision unit program. | Purposive Sample of clinical and administrative staff from seven participating sites. | 31 clinical and administrative staff from 7 sites were recruited. 24 were interviewed in phase 1 26 in phase 11 and 19 in both phases. Participants varied across sites but included ED physicians, clinical directors, nursing staff, IT decision support staff and senior hospital administrators. | Barriers and facilitators to the implementation of this program were identified. Team building and stakeholder involvement, use of clinical care protocols and involvement of specialist services facilitated the process. Poor communication and difficulty in making decisions were barriers. | High |
| Sampson et al (2019)  UK  Article No 23 | Multiple case design using qual research in 3 EDs in the UK. Case studies incorporated 143 hours of non-participant observation, documentary analysis and semi-structured interviews with 36 staff. | Thematic Analysis | The aim was to understand how pain scores are used in practice and the mechanisms by which pain scoring may influence pain management. | Purposive Sample of clinical staff. | 20 emergency physicians and 16 nurses were interviewed to ascertain their experience of pain scoring in the ED. | ED staff used the pain score for 2 purposes as an auditable tool for guiding patient management and as a tool to monitor patient experience. This led to bias in scoring which was a barrier. | High |
| Schoenfeld et al (2019)  USA  Article No 24 | One on one semi-structured interviews with ED physicians. | Thematic Analysis using the Dedoose qualitative data management system. | The aim was to understand when and why ED Physicians engage in shared decision-making. Barriers and facilitators to the process will also be identified. | Purposive sample of attending ED physicians. 15 practicing ED physicians were interviewed to ascertain their experience in using shared decision-making. | Participant were stratified by gender, years in practice since residency, region of primary employment, academic vs community practice setting and location of training. | Barriers were identified such as a lack of follow up, challenges of communicating uncertainty. Local culture also impacted on practices as well as time constraints, fear and uncertainty. | Moderate |
| Skyttberg et al (2016)  Sweden  Article No 25 | One on one semi-structured interviews. | Content Analysis | The study aims to explore the factors affecting vital sign data quality in Swedish EDs from the perspective of ED physicians. A further aim is to ascertain how vital sign quality can be improved. | Sixteen physicians and nurses from nine hospitals. Follow-up interviews were also conducted to validate data. | Participants were required to be registered as a nurse or a doctor for a minimum of 5 years or experience in emergency care in particular triage and vital sign documentation. | Poor documentation practices resulted in low currency, completeness and interoperability of the vital signs. The process was found to require standardisation and improved documentation, supports and auditing. | High |
| Tarrant et al (2016)  UK  Article No 26 | Ethnographic Study. 300 hours of non-participant observation in EDs and acute receiving units. Interviews with 43 hospital staff across six pilot sites. One on one semi-structured interviews. | Data analysis using a constant comparative approach. | To explore frontline practice in implementing the Sepsis-Six and to identify barriers to reliable implementation of bundle components. | Purposive sample across six sites, interviews were conducted with ED and Acute Receiving Unit Staff along with direct observation of practices. | 300 Hours of Direct Observation and 43 interviews (Purposive Sample with Staff at each site) Consultants, Doctors, Senior Nurses, Junior Nurses, Managers, Trainee Doctors, Pharmacists or varying levels of experience and differing roles. | Implementation efforts that focus on individual behaviour change to improve uptake of the Sepsis Six should be supplemented by an understanding of the bundle as a complex trajectory of work in which improving reliability requires attention to coordination of workflow, as well as addressing the mundane problems of interruptions and operational failures that obstruct task completion. | High |
| Tavender et al (2014)  Australia  Article No 27 | One on one semi-structured interviews with doctors, nurses and directors. | Iterative process of transcript analysis and open-coded text. These were then coded to domains in the Theoretical Domains Framework | The aim was to identify and explore the factors that influence the uptake of four key evidence based recommended practices for managing mild-traumatic brain injury. | Stratified purposive sample of clinicians from small to large metropolitan, inner and outer regional EDs to ensure all viewpoints were represented. Saturation reached at 42 participants. | 42 Participants (9 Directors, 20 Doctors and 13 Nurses) were interviewed over a seven month period. | The results suggested that the prospective assessment and identification of post-traumatic amnesia was influenced by knowledge; beliefs about consequences; environmental context and resources; skills; social/professional role and identity; and beliefs about capabilities. Barriers and facilitators to the process were clearly identifiec. | High |
| Van Der Wulp et al (2011)  Netherlands  Article No 28. | One on one semi-structured interviews. | Interviews were transcribed for further analysis with Weft QDA. With this program text parts were labelled and ordered. | Exploration of ED nurses' motivation/  subjective experiences pertaining to pain assessment at triage. | Purposive sample of 13 ED/Triage Nurses. | Characteristics of participants Age, Gender, Specialist Training, ED Experience and Experience with Screening and Assessment were recorded. | Clear rationale for ED nurses' not undertaking pain assessment as per guidelines. Areas for further study and exploration indicated, Implications for practice and the use of electronic health records to induce assessment were discussed and the adaptation/updating of guidelines was also indicated. Evaluation of the triage process to streamline assessment was also discussed. | Moderate |
| Wolf et al (2019)  USA  Article No 29 | Semi-Structured Qualitative Focus Groups were conducted after a quantitative survey to expand and build upon data. The qualitative data was used for this review | Qualitative Content Analysis | The aim of the study is to explore the perception of emergency nurses' perceptions of their ability to care for geriatric patients in the emergency setting. | National conference for emergency nurses. 23 participants recruited on the day of the conference comprising of two focus groups. All consenting participants were included in the focus groups on the day of the conference. | Purposive and diverse sample recruited from a pool of emergency nurses via e-mail. Demographics Age, Gender, Country of Origin. Specific nursing role, level of experience, Education, Facility type and ED patient population all outlined. | Barriers and facilitators to care provision, areas for expanded practice and interventions to improve care within the ED are identified and detailed.  Specific deficits in geriatric-specific screenings, accommodations and communications were identified. | High |
| Eagles et al (2022)  Canada  Article No 30 | One on one semi-structured face-to-face interviews. | Published TDF methods served as a guide for the three stages of analysis: (i) coding utterances from interviews into theoretical domains; (ii) generating specific belief statements and (iii) identifying relevant and non-relevant domains. | The aim was to identify barriers and facilitators to delirium screening by nurses in older ED patients. | Purposive sample: 15 Interviews were conducted with ED nurses. | Bedside nurses, nurse educators, nurse managers and specialised geriatric emergency management nurses who worked in the EDs were invited to participate in the study. To get maximum variation in responses, they purposively selected, of those whom responded, interviewees with varying roles (bedside, managerial and educational), gender and clinical experience. | Three dominant themes emerged: (i) lack of clinical prioritisation because of competing demands, lack of time and heavy workload; (ii) discordance between perceived capabilities and knowledge and (iii) hospital culture. | High |
