# Supplementary material for: A qualitative evidence synthesis (QES) exploring the barriers and facilitators to screening in emergency departments using the theoretical domains framework
Source: BMC Health Serv Res. 2023 Oct 11;23:1090. doi: 10.1186/s12913-023-10027-3 (PMC10568862; doi:10.1186/s12913-023-10027-3)
Supplement: Supplementary file 5 — Additional file 5: Supplementary File 5. Theoretical Domains Framework Atkins 2017 Definitions and Constructs Table. [file 12913_2023_10027_MOESM5_ESM.docx]

| **Supplementary file 5:** Theoretical Domains Framework Atkins 2017 Definitions and Constructs Table  Best-Fit Framework Synthesis (BFFS) produces conceptual models to assist in explaining and describing the health behaviours or decision-making of patients or other groups by using a transparent and pragmatic process (Dixon-Woods, 2011). To facilitate the synthesis of primary research, using this method, an *a priori* framework was identified to facilitate the evidence synthesis. This *a priori* framework was based on the definitions and component constructs of the Theoretical Domains Framework (TDF) outlined by Atkins et al (2017). | |
| --- | --- |
| **Domain (Definitions)** | **Constructs** |
| Knowledge(An awareness of the existence of something) | - Knowledge (including knowledge of condition/scientific rationale). - Procedural knowledge. - Knowledge of task environment. |
| Skills(An ability or proficiency acquired through practice) | - Skills/Skills Development - Competence/Ability - Interpersonal skills - Practice/Skill assessment |
| Social/professional role and identity(A coherent set of behaviours and displayed personal qualities of an individual in a social or work setting) | - Professional identity - Professional role - Social identity/Identity - Professional boundaries - Professional confidence - Group identity - Leadership - Organisational commitment |
| Beliefs about capabilities(Acceptance of the truth, reality or validity about an ability, talent or facility that a person can put to constructive use | - Self-confidence - Perceived competence - Self-efficacy - Perceived behavioural control - Beliefs/Self-esteem - Empowerment - Professional confidence |
| Optimism (The confidence that things will happen for the best or that desired goals will be attained | - Optimism - Pessimism - Unrealistic optimism - Identity |
| Beliefs about Consequences(Acceptance of the truth, reality, or validity about outcomes of a behaviour in a given situation) | - Beliefs - Outcome expectancies - Characteristics of outcome expectancies - Anticipated regret - Consequents |
| Reinforcement(Increasing the probability of a response by arranging a dependent relationship, or contingency, between the response and a given stimulus) | - Rewards (proximal/distal, valued/not valued, probable/improbable) - Incentives - Punishment - Consequents - Reinforcement - Contingencies - Sanctions |
| Intentions(A conscious decision to perform a behaviour or a resolve to act in a certain way) | - Stability of intentions - Stages of change model - Transtheoretical model and stages of change |
| Goals(Mental representations of outcomes or end states that an individual wants to achieve) | - Goals (distal/proximal) - Goal priority - Goal/target setting - Goals (autonomous/controlled) - Action planning - Implementation intention |
| Memory, attention and decision processes(The ability to retain information focus selectively on aspects of the environment and choose between two or more alternatives) | - Memory - Attention - Attention control - Decision making - Cognitive overload/tiredness |
| Environmental context and resources(Any circumstance of a person’s situation or environment that discourages or encourages the development of skills and abilities, independence, social competence and adaptive behaviour) | - Environmental stressors - Resources/material resources - Organisational culture/climate - Salient events/critical incidents - Person × environment interaction - Barriers and facilitators |
| Social influences(Those interpersonal processes that can cause individuals to change their thoughts, feelings or behaviours) | - Social pressure - Social norms - Group conformity - Social comparisons - Group norms - Social support - Power - Intergroup conflict - Alienation - Group identity - Modelling |
| Emotion (A complex reaction pattern involving experiential, behavioural, and physiological elements, by which the individual attempts to deal with a personally significant matter or event) | - Fear - Anxiety - Affect - Stress - Depression - Positive/negative affect - Burn-out |
| Behavioural regulation(Anything aimed at managing or changing objectively observed or measured actions) | - Self-monitoring - Breaking habit - Action planning |

Atkins, L., Francis, J., Islam, R., O’Connor, D., Patey, A., Ivers, N., Foy, R., Duncan, E.M., Colquhoun, H., Grimshaw, J.M., Lawton, R. and Michie, S. (2017) A guide to using the Theoretical Domains Framework of behaviour change to investigate implementation problems, Implementation Science, 12:77, DOI 10.1186/s13012-017-0605-9 Pages 4 and 5 Table 1.
