# Supplementary material for: A qualitative evidence synthesis (QES) exploring the barriers and facilitators to screening in emergency departments using the theoretical domains framework
Source: BMC Health Serv Res. 2023 Oct 11;23:1090. doi: 10.1186/s12913-023-10027-3 (PMC10568862; doi:10.1186/s12913-023-10027-3)
Supplement: Supplementary file 6 — Additional file 6: Supplementary file 6. Sample of Coding Excerpts from Article No 13 Menser et al (2015) Organised under Domains with Related Constructs. [file 12913_2023_10027_MOESM6_ESM.docx]

**Supplementary file 6: Sample of Coding Excerpts from Article No 13 Menser et al (2015) Organised under Domains with Related Constructs.**

**Environmental Context and Resources**

No 13 Pg 126 Menser et al (2015) The program, as implemented, aligned with initial program goals, but it was dependent on ED screening staff and RHC (Rural Health Clinic) availability. Some adjustments to the program were needed, including RHC hours, consistency among staff in making referrals, patient education, and improving patient uptake on the referral **Vital Practice Adaptations, Resources.**

No 13 Pg 126 Menser et al (2015) Stakeholders reported lessons learned related to training, staff buy-in, Emergency Medical Treatment and Labour Act (EMTALA), and intra-organizational cooperation. **Organisational Culture and Adaptations.**

No 13 Pg 126 Menser et al (2015) The other RHC was open from Monday to Friday

from 8:00 a.m. to 5:00 p.m.; hours at this location were not extended due to lack of available providers and proximity of the other clinic. **Barriers to the Screening Process and Associated Referrals.**

No 13 Pg 129 Menser et al (2015) Despite the fact that the screening and referral protocol has been articulated clearly, the process is still subjective. There was some indication that fidelity was not only correlated to the individual performing the MSE, but also whether the

RHC was open at the time of screening. One respondent stated, in contrast to the protocol, “*After hours we normally do not screen a patient.” “It is not really possible*

*to change organizations in any truly meaningful sense unless organizational participants perform their jobs differently, change their thinking or attitudes in ways that support the needed changes, and so on.”* **Organisational Commitment, Person X Environment, Barriers.**

No 13 Pg 131 Menser et al (2015) The conversion rate was defined by the organization as

the percent of patients screened out of the ED and referred to the RHC that seek care at the RHC. Respondents reported 2 main factors affecting patients’ choice to seek care from the RHC once referred from the ED—financial responsibility and ease of access to the RHC. At the RHC, patients were asked for payment upon arrival by either cash or credit card. The new up-front financial responsibility was noted as a barrier to RHC use for some referred patients who would not be asked for an up-front payment in the ED. However, system and RHC administrators reported that patients with private insurance were often pleased with the smaller out-of-pocket expense obligation for a primary care visit (often $30-$45) in the RHC

compared to the higher charges by the ED (often a minimum $100 co-payment plus coinsurance). **Results of Screening, Barriers to Referral from the ED.**

No 13 Pg 131 Menser et al (2015) The physical proximity of the RHC in relation to the ED varied by site, but in both cases was co-located in the same building as the ED. Respondents echoed the notion that close proximity of the RHC to the ED was an important factor for

patient access and convenience, and, thus, was cited by respondents as increasing the chance of a given patient’s conversion to RHC care. **Referral Infrastructure/Proximity.**

No 13 Pg 132 Menser et al (2015) System leadership confirmed that 4 investigations related to EMTALA (Emergency Medical Treatment and Labour Act) concerns were conducted in relation to the program, but that all 4 investigations noted that the organization, medical screening protocol, and referral initiative were in full compliance with EMTALA provisions **Legal Support/Infrastructure to Support Screening.**

No 13 Pg 132 Menser et al (2015) Transferring records between departments helps to avoid redundancy, increasing efficiency. The ED staff was given access to the scheduling system to make RHC appointments and the RHC staff track the disposition of referred patients and routinely communicate the conversion rate with ED staff. **Resources, Follow-up**

No 13 Pg 132 Menser et al (2015) Hospitals systems often implement several changes in

tandem; the organization studied was no exception. Thoughtful coordination of change is essential to yield best possible outcomes. A new electronic health record (EHR) was implemented in parallel to the MSE and referral initiative. The learning curve that the new EHR required coupled with the adoption of the new RHC referral protocol sometimes reduced the pace of ED care. One respondent said, “The ER was actually on a paper record

that was scanned in and retained electronically, but they went full electronic—physician order entry, documentation, everything electronic . . . .” Future implementation of this type of program should consider other large-scale organizational change initiatives in progress and plan or adjust patient and staff expectations accordingly. **Planning for Organisational Change.**

No 13 Pg 132 Menser et al (2015) A report of follow-up calls by the RHC with patients who were referred but did not seek RHC care indicated that many patients had access to other community providers, but were seeking ED care due to convenience or uncertainty regarding the severity of their condition. **Barriers of the Referral Process.**

No 13 Pg 133 Menser et al (2015) It is likely that some uninsured patients classified as non-emergent through the MSE will find the out-of-pocket cost of seeking nonemergency care at the RHC prohibitive. This financial barrier could decrease access to nonemergency care for those patients and lead to reduced outcomes and lower satisfaction for some segments of the

population. **Process Barriers.**

No 13 Pg 133 Menser et al (2015) However, for those who establish a new medical home with the RHC, population health would likely improve through better care monitoring and other benefits of regular primary care services. Respondents generally reported that the MSE has been financially beneficial to both the organization and its patients. One respondent

stated, *“Allocating resources where they most are needed is so important for success.”* Similarly, another respondent remarked, *“Our purpose is to see everybody, but also*

*we have to be financial stewards for our system to be able to provide great care for everybody.”* **Environmental Resources and Benefits.**

**Knowledge**

No 13 Pg 127 Menser et al (2015) The health system has a specific protocol to medically

screen all patients presenting to the ED for care. There is no charge for the MSE, and it is conducted by a registered nurse (RN) with special training needed to document patient care needs and acuity using the Emergency Severity Index (ESI). The ESI score is assigned by the RN after he or she has assessed the patient’s chief complaint, vital signs, and both the appearance and mental fitness of the patient **Procedural Knowledge, Knowledge of Task Environment.**

No 13 Pg 127 Menser et al (2015) When a patient presents to the ED, he or she signs in with the receptionist and completes the intake forms. The patient is taken to a triage room where an RN assesses the patient’s condition, including vital signs, chief complaint, and brief medical history. Patients with high acuity (ie, ESI 1-3) or that are categorically ineligible

for RHC referral proceed to the ED for care. Patients who do not require emergency care and who are categorically eligible for RHC referral are provided ED discharge instructions along with information about care available to them through the RHC **Procedural Knowledge, Knowledge of the Screening Process, Rationale.**

No 13 Pg 131 Menser et al (2015) One respondent said, *“I think the main thing is to have*

*a strong training program . . . that’s the core of the process. If right off, you answer [RNs’] questions, you give them the necessary tools, and they’ll be able to implement*

*it right.” Upon successful completion of the training program, all RNs are expected to share the responsibility of conducting the MSE. One respondent said, “It’s been good that it’s not a certain person that’s on duty that has to do the screens one after the other all day long. It’s*

*whoever’s available to do it right then.”* **Knowledge Development, Staff Supports, Peer Development.**

No 13 Pg 131 Menser et al (2015) RNs reported impressions that the patient community had gained understanding of the new program during its inaugural year. *“Getting people to a provider for continuation of care throughout their life is great for the public. So many people*

*just didn’t have a doctor so they just showed up at the ER with their cold.”* Another respondent stated, *“It’s very good to see repeat business from an emergency room visit*

*that goes into a clinic and [patients are] establishing a relationship* [with the RHC].” **Knowledge about the Referral Process and Available Resources.**

**Social Influences**

No 13 Pg 126 Menser et al (2015) Stakeholders reported lessons learned related to training, staff buy-in, Emergency Medical Treatment and Labour Act (EMTALA), and intra-organizational cooperation. **Social Norms and Staff Supports.**

No 13 Pg 132 Menser et al (2015) The leadership of the health system encouraged patient

education regarding the program and offered full support of the initiative and its staff in these situations, which eased the initial concerns of RNs. **Leadership**

No 13 Pg 132 Menser et al (2015) The success of the MSE and RHC referral initiative has

required extensive cooperation between the ED and the RHC staff. Though these collaborative working relationships predated the implementation of the MSE screening

and referral initiative, the new program has strengthened their level of cooperation. **Interdisciplinary Collaboration.**

No 13 Pg 133 Menser et al (2015) The ultimate goal of most system-wide procedural change is that both staff and patients fully accept the new initiatives as such adaptation is essential to the sustainability of any new program or process. As such, a more detailed study of community acceptance strategies may also be needed. **Social Norms and Acceptance.**

**Goals**

No 13 Pg 126 Menser et al (2015) The system was able to leverage excess capacity of affiliated RHCs to accommodate low-acuity patients referred from the ED and may lead to improvements in Triple Aim goals of increased patient satisfaction, better population health and outcomes, and lower per capita costs. **Goal Setting/Targets.**

No 13 Pg 130 Menser et al (2015) Both sites reported that nonemergency patients sometimes reacted with surprise and were upset when they were informed that they would not be seen by an ED physician. One respondent said, *“If you just take a few minutes to educate [patients] on clinic hours and what the process does for them, people are usually thankful. Their co-pays are so much less for a clinic visit versus an ER visit.”* Another respondent commented, “*We just have to keep educating them, and communication is the number one thing. When they’re here in the clinic and the docs see them, they encourage them if it’s an emergency, present to the ER. But if it’s not, then definitely come to the clinic and let us see you [at the* RHC].” **Goal Setting/Targets, Goals of the Screening/Referral Process.**

No 13 Pg 130 Menser et al (2015) The ED and RHC employees reported that they work together to provide the best possible care coordination. One respondent said, “I’m extremely

proud of this program. I think it has been great for our hospital, for our community, and for our patients.” **Goal Setting.**

No 13 Pg 132 Menser et al (2015) Hospitals systems often implement several changes in

tandem; the organization studied was no exception. Thoughtful coordination of change is essential to yield best possible outcomes. A new electronic health record (EHR) was implemented in parallel to the MSE and referral initiative. The learning curve that the new EHR required coupled with the adoption of the new RHC referral protocol sometimes reduced the pace of ED care. One respondent said, “The ER was actually on a paper record

that was scanned in and retained electronically, but they went full electronic—physician order entry, documentation, everything electronic . . . .” Future implementation of this type of program should consider other large-scale organizational change initiatives in progress and plan or adjust patient and staff expectations accordingly. **Goal Setting, Implementation Intention.**

No 13 Pg 132 Menser et al (2015) Additional studies are needed to identify specific short-term and long-term impacts of the program on the Institute for Healthcare Improvement (IHI) Triple Aim goals related to improving the patient experience (e.g., quality and satisfaction), improving the health of populations, and reducing the per capita cost of care.16 However,

respondents provided some insights to Triple Aim goals. Specifically, *satisfaction* with care is likely to improve with reduced ED congestion due to nonemergency use. **Goals of the Screening/Referral Process.**

**Professional Role and Responsibility**

No 13 Pg 127 Menser et al (2015) The health system has a specific protocol to medically

screen all patients presenting to the ED for care. There is no charge for the MSE, and it is conducted by a registered nurse (RN) with special training needed to document patient care needs and acuity using the Emergency Severity Index (ESI). The ESI score is assigned by the RN after he or she has assessed the patient’s chief complaint, vital signs, and both the appearance and mental fitness of the patient. **Professional Role, Functions of the Role in the Screening Process.**

No 13 Pg 127 Menser et al (2015) When a patient presents to the ED, he or she signs in with the receptionist and completes the intake forms. The patient is taken to a triage room where an RN assesses the patient’s condition, including vital signs, chief complaint, and brief medical history. Patients with high acuity (i.e., ESI 1-3) or that are categorically ineligible

for RHC referral proceed to the ED for care. **Professional Role, Screening Process in the ED.**

No 13 Pg 128 Menser et al (2015) Patients referred to the RHC for nonemergency care receive assistance from ED staff in scheduling a short-notice RHC appointment and locating the RHC. Patients checking with the RHC receptionist and complete the required intake forms, and they can complete their clinic visit at the RHC, unless, at any point, RHC clinicians identify an emergency care need requiring referral back to the ED. **Multidisciplinary Roles in the Screening Process.**

No 13 Pg 129 Menser et al (2015) Another factor that was not carried out as planned was

consistency in medical screening and RHC referral among RNs. Intervention fidelity can be defined as “*the adherent and competent delivery of an intervention.”*13(p.63). Three separate interviewees volunteered that consistency among RN screeners varied greatly. One respondent stated that *“There’s still some resistance [to RHC referrals]. . . [RNs who refer to the RHC the least] just go ahead and bring [patients] back [to the ED], because they*

*don’t want to make people unhappy.”* **Professional Role/Responsibility/Competence.**

No 13 Pg 131 Menser et al (2015) **“***Upon successful completion of the training program, all RNs are expected to share the responsibility of conducting the MSE. One respondent said, “It’s been good that it’s not a certain person that’s on duty that has to do the screens one after the other all day long. It’s whoever’s available to do it right then.”* **Role in Knowledge/Peer Development.**

No 13 Pg 131 Menser et al (2015) Nurse acceptance of the new RHC referral responsibility required strong support from local and system administration, which respondents indicated was particularly evident after initial patient complaints were received **Organisational Commitment and Supports.**

No 13 Pg 131 Menser et al (2015) Since only non-emergent (i.e., ESI 4 or 5) patients were eligible for referral from the ED to the RHC, it became the RNs’ responsibility to explain to these patients that they did not require emergency care. One respondent said that EMTALA was also an initial concern of the physician group due to possible legal repercussions. **Professional Responsibilities.**

No 13 Pg 132 Menser et al (2015) The leadership of the health system encouraged patient

education regarding the program and offered full support of the initiative and its staff in these situations, which eased the initial concerns of RNs. **Organisational Commitment.**

**Reinforcement**

No 13 Pg 129 Menser et al (2015) The extension of RHC hours changed from initial planning

to the time of interview, a year later. In 1 location, the health care system advertised extended RHC hours in both print and radio media to increase community awareness. The clinic’s extended hours were 7 days a week from 8:00 a.m. to 8:00 p.m. However, that strategy

was revised*. “We . . . just weren’t seeing enough [patients] on Sundays and Saturdays for it to be cost effective . . . .We just weren’t getting that many [medically screened out individuals and other patients] that couldn’t wait until Monday.”* One respondent stated, *“[the RHC*

*referral program has] extended clinic hours, and so people now know—it’s getting more commonly known in the community—that they don’t have to even come to the ER anymore. They know the clinic’s open after hours and they can go straight over there****.****”* Hours at one location were 8:00 a.m. to 8:00 p.m. Monday to Friday, and 8:00 a.m. to 4:00 p.m. on Saturdays at the time of the interview. **Contingencies, Planning, Adapting the Screening Process.**

No 13 Pg 131 Menser et al (2015) One RHC manager reported a strategy of follow-up calls within 24 hours to patients referred from the ED to ensure that they were able to make an appointment, if desired. Some appealing aspects of this program for RHC buy-in included the

ability to expand staffing and provider capacity, and establish a new medical home for converted patients and their families or others referred by them. **Contingencies.**

No 13 Pg 132 Menser et al (2015) Transferring records between departments helps to avoid redundancy, increasing efficiency. The ED staff was given access to the scheduling system to make RHC appointments and the RHC staff track the disposition of referred patients and routinely communicate the conversion rate with ED staff. **Reinforcement.**

No 13 Pg 132 Menser et al (2015) However, the financial environment of the relatively large regional rural health system studied may not match that of a stand-alone hospital without a nearby, affiliated RHC. In particular, rural stand-alone facilities may have patient volume, capacity, and/or payment structures that can accommodate a mix of emergent, urgent, and non-urgent patients in the ED environment. These facilities may consider adopting other strategies such as triage protocols to improve care efficiency. **Contingencies, Process Adaptations.**

**Behavioural Regulation**

No 13 Pg 129 Menser et al (2015) Another factor that was not carried out as planned was

consistency in medical screening and RHC referral among RNs. Intervention fidelity can be defined as “*the adherent and competent delivery of an intervention.”*13(p.63). Three separate interviewees volunteered that consistency among RN screeners varied greatly. One respondent stated that *“There’s still some resistance [to RHC referrals]. . . [RNs who refer to the RHC the least] just go ahead and bring [patients] back [to the ED], because they*

*don’t want to make people unhappy.”* Despite the fact that the screening and referral protocol has been articulated clearly, the process is still subjective. There was some indication that fidelity was not only correlated to the individual performing the MSE, but also whether the

RHC was open at the time of screening. One respondent stated, in contrast to the protocol, “*After hours we normally do not screen a patient.” “It is not really possible*

*to change organizations in any truly meaningful sense unless organizational participants perform their jobs differently, change their thinking or attitudes in ways that support the needed changes, and so on.”* **Breaking Habit, Action Planning.**

No 13 Pg 130 Menser et al (2015) Both sites reported that nonemergency patients sometimes reacted with surprise and were upset when they were informed that they would not be seen by an ED physician. One respondent said, *“If you just take a few minutes to educate [patients] on clinic hours and what the process does for them, people are usually thankful. Their co-pays are so much less for a clinic visit versus an ER visit.”* Another respondent commented, “*We just have to keep educating them, and communication is the number one thing. When they’re here in the clinic and the docs see them, they encourage them if it’s an emergency, present to the ER. But if it’s not, then definitely come to the clinic and let us see you [at the* RHC].” **Breaking Habit, Action Planning.**

No 13 Pg 131 Menser et al (2015) There was some noted apprehension by the nursing staff

in the infancy of the program’s implementation related to changing how RNs screen patients during the ED intake process. Nurses were accustomed to providing care to anyone who presented at the ED; deciding that a patient’s care needs were non-emergent and communicating that information to the patient was not a comfortable change for all RNs. **Breaking Habits, Staff Buy-in.**

No 13 Pg 131 Menser et al (2015) Physicians may have initially been concerned with the increasing authority of RNs, but they have found that the RHC referral initiative allowed them to have *“more time with the patients that they needed to spend time with.”* Another respondent said, *“Nurses actually feel, because of this process, that they are able to take care of the sicker patients that need the care. They’re able to give them more of their time instead of just run, run, run, run.”* Buy-in of RHC employees was also a critical component to the success of this initiative; they adjusted their care processes and staffing to accommodate short-notice appointments, shared the appointment scheduling system with the ED, and managed disgruntled patients who were sometimes just screened out of the ED and asked to complete similar. **Staff Buy-in, Behavioural Adaptation.**

No 13 Pg 133 Menser et al (2015) Examining the screening patterns of different personnel

would be beneficial to determine the fidelity of the initiative and the staff’s level of consistency and buy-in. **Action Planning, Staff Buy-in and Behaviours.**

**Skills**

No 13 Pg 130 Menser et al (2015) The process of patient education was an essential element to the success of the MSE initiative. Ideally patients referred to the RHC would understand they were not being turned away from receiving medical care, but that the organization was offering them the option of care in a more appropriate setting. **Educational Skills**

No 13 Pg 130 Menser et al (2015) Based on state regulations, emergency medicine physicians must train RNs on how to conduct the MSE and assign ESI scores. The standardized training is offered frequently to minimize any impacts of nurse turnover, lasts

4 days, and covers a variety of clinical scenarios. It also includes a post-training assessment to ensure each nurse has all the skills necessary to properly perform the MSE. **Skills Development and Assessment.**

**Beliefs about Consequences**

No 13 Pg 132 Menser et al (2015) Physicians were also concerned about how diverting patients from the ED would affect them. One respondent stated, *“The ED docs were reluctant because they were not involved in the process. They just didn’t know how it would affect the ED numbers overall. Now it’s just part of daily work flow*.” **Apprehension, Possible Consequents.**

No 13 Pg 131 Menser et al (2015) Respondents reported that several patients who were accustomed to being seen in the ED expressed concerns that they were being denied emergency care, which would violate the Emergency Medical Treatment and Active

Labour Act (EMTALA) requirement that medical care be provided to all persons who present at an ED with an emergent condition.15 Since only non-emergent (ie, ESI 4 or 5) patients were eligible for referral from the ED to the RHC, it became the RNs’ responsibility to explain to these patients that they did not require emergency care. One respondent said that EMTALA was also an initial concern of the physician group due to possible legal repercussions. **Consequences of the Screening/Referral Process, Management of the Consequents.**

No 13 Pg 132 Menser et al (2015) Many respondents emphasized an ultimate goal to improve

the quality of care delivered to patients, and thus impact *population health and outcomes*. One of the main concerns initially expressed by RNs charged with implementing the MSE was that patients referred to the RHC might forgo medical care until the condition became

emergent. A respondent said, *“[The nurses] just wanted to make sure that these folks, when they were leaving, were actually getting to the clinic*.” **Fears about Impact of the Process.**

No 13 Pg 133 Menser et al (2015) A program designed to medically screen and refer

nonemergency patients from the ED to another care setting is particularly beneficial for rural areas since there are significant differences not only in the number and type of providers practicing in rural areas, but also in the demographics of rural residents, both of which can accentuate problems related to ED overcrowding. Patients in rural areas compared to urban areas are generally less likely to have insurance, have lower self-reported health status, and have reduced access to primary care providers. **Urban vs Rural Processes and Consequents.**

**Beliefs about Capabilities**

No 13 Pg 131 Menser et al (2015) Physicians may have initially been concerned with the increasing authority of RNs, but they have found that the RHC referral initiative allowed them to have *“more time with the patients that they needed to spend time with.”* Another respondent said, *“Nurses actually feel, because of this process, that they are able to take care of the sicker patients that need the care. They’re able to give them more of their time instead of just run, run, run, run.”* Buy-in of RHC employees was also a critical component to the success of this initiative; they adjusted their care processes and staffing to accommodate short-notice appointments, shared the appointment scheduling system with the ED, and managed disgruntled patients who were sometimes just screened out of the ED and asked to complete similar forms and pay up front for RHC care. **Capabilities of the Screening Process.**

No 13 Pg 132 Menser et al (2015) Findings from this formative evaluation suggest that the

MSE and referral initiative was successful in achieving early implementation and operational goals. Non-emergent patients were being referred to a more appropriate care setting, which was decongesting the EDs in these small rural facilities. Rerouting patients to affiliated RHCs

may also enhance financial performance in an environment of increasingly limited hospital margins. **Capabilities of the Process.**

No 13 Pg 132 Menser et al (2015) This is because the entire volume of ED admissions to inpatient care in the rural facilities would be from those with emergency conditions (i.e., ESI 1-3) and also because patients referred to the affiliated RHC were responsible for payment

or co-payment at the time of service. **Capabilities of the Process.**

No 13 Pg 130 Menser et al (2015) Through the initiative, the RHC hours were extended,

increasing convenience for non-emergent medical care. One respondent said, *“We [are] able to concentrate on giving services to those who really need emergency services as opposed to those who just go [to the ED] for sniffles, work excuse[s], or just a prescription refill, which*

*all those can be done in the clinical setting [RHC].”* Interviewees did suggest that patients were beginning to utilize the RHC for primary care and that staffing and resources for the RHCs were being adjusted to reflect a higher demand for primary care services. They also reported that patients initially referred from the ED to the RHC had started to refer their family members and friends to the clinic as well, suggesting both acceptance and satisfaction

with care. One respondent said, *“I think the early success is that [the MSE] puts the patient in an environment where they actually get the treatment that they need quicker.”* **Capabilities, Benefits of the Process.**

**Optimism**

No 13 Pg 130 Menser et al (2015) The ED and RHC employees reported that they work together to provide the best possible care coordination. One respondent said, “I’m extremely

proud of this program. I think it has been great for our hospital, for our community, and for our patients.” **Optimism**
