# Supplementary material for: A qualitative evidence synthesis (QES) exploring the barriers and facilitators to screening in emergency departments using the theoretical domains framework
Source: BMC Health Serv Res. 2023 Oct 11;23:1090. doi: 10.1186/s12913-023-10027-3 (PMC10568862; doi:10.1186/s12913-023-10027-3)
Supplement: Supplementary file 7 — Additional file 7: Supplementary file 7. Findings Categorised under TDF Domains. [file 12913_2023_10027_MOESM7_ESM.docx]

**Supplementary file 7 Findings Categorised under TDF Domains.**

**The numbers in brackets behind each finding relate to the numbering of included studies in the table of articles in Supplementary File Four.**

**Domain:** Knowledge, Behavioural Regulation

**Finding 1:** HCWs experience illustrates that procedural knowledge and an awareness of knowledge deficits can impact on the screening and referral process.

HCWs required in-depth knowledge of the screening and referral process (2, 7, 27, 28). This *“procedural knowledge”* included knowledge of the screening tool, referral pathways, associated guidelines or protocols and the ED environment (1, 2, 4, 7, 16, 27, 28). Staff relied on this knowledge to underpin their screening practices and were mindful of their responsibility to update their practice accordingly (7, 24). For example, when screening patients for falls risk (7) and assessing their pain (24), HCWs determined the appropriate care pathway post fall and the appropriate level of analgesia (7, 24). In instances where screening processes were not adhered to, it was due to a lack of knowledge regarding guidelines around care or a lack of screening early in their admission to the ED (e.g. during triage) (9, 29).

HCWs experience also indicates that although screening may appear straightforward, this process often involved multiple staff members and included multiple interdependent tasks (2, 9, 13, 21 27). Some aspects required prioritisation, scheduling between staff and complex referral pathways (2, 9, 13, 27). For example, sepsis screening involved the wider multidisciplinary team (ED Physicians, Nurses, Critical Care Specialists) and multiple interventions were employed (Screening, Phlebotomy, Scans) (9). Staff were knowledgeable around these complex processes with many discussing them in-depth (2, 13, 21, 22, 27). However, education, time and experience were required to attain this level of knowledge (2, 12, 27). Due to this, new and junior members of staff found it difficult to comprehend and orientate themselves to multiple screening and referral processes within ED settings (2, 7, 9, 21, 28). Consequently, they failed to recognise the potential impact of inaccurate or omitted screening (2, 7, 9, 21, 28).

HCWs also experienced a lack of procedural knowledge around who to liaise or consult with during the screening/referral process and how to address knowledge deficits (19, 22, 28). For example, when required to liaise with neurology, junior doctors were unsure of which consultant to discuss the case with, how to refer the patient and who to refer them to (22, 28). Furthermore, ED staff needed to initiate their own education around screening (4, 22, 28). ED physicians and nurses reported being aware of the guidance that underpinned screening, but admitted they needed to inform their practice by studying these guidelines (4, 22, 28).

**Domain:** Knowledge

**Finding 2:** HCWs perceived a lack of knowledge among ED staff and their peers pertaining to screening processes in the ED.

ED consultants, registrars and nurses expressed concern that more complex forms of screening, for example for post traumatic amnesia (PTA), were performed by staff with limited knowledge of what the screening tool was for and how to accurately use it (28). ED staff were often unsure of why they were screening patients (1, 5, 12, 21, 28). Education was deemed vital to address this knowledge deficit and attain staffs’ commitment to the screening process (5, 21). A lack of standardisation of the screening process, probably stemming from the lack of clear written policies and training, underpinned the lack of knowledge (1, 28).

HCWs experienced a lack of knowledge about the availability of validated tools to screen patients (9, 12, 28). ED staff who were aware of these tools were unsure of how to access or use them within the ED (9, 12, 28). ED consultants, registrars and junior doctors were unaware of the resources available to them to support the screening process which included guidelines, information leaflets and algorithms (5, 12, 28). Consequently, they were unable to accurately screen for PTA and falls risk and designate care in an evidence-based manner (5, 12, 28). Ready access to a variety of resources and education was identified as an enabler of screening use within the ED, even if staff lacked in-depth knowledge (2, 22). ED staff also experienced a lack of procedural knowledge among more senior members of staff who did not know what screening involved or what its purpose was (21, 22).

**Domain:** Skills, Behavioural Regulation

**Finding 3:** HCWs developed skills to engage in the screening and referral process competently, these skills were attained through practice-based experience, educational opportunities and skills assessment.

**Skill Development**

Having clear, easy and accessible screening and referral processes were facilitators to skills development (2, 4). Consequently, ED staff could easily identify the skills they required to screen and refer patients (2, 4). To assist in skills development, the provision of appropriate theoretical and practice-based skills training was considered necessary by staff (16, 19, 21), particularly for specific conditions such as trauma, sepsis and acute cardiac conditions (9, 19, 22). Didactic and simulation-based techniques were essential to ensure screening uptake and usage (16, 19, 21). Staff turnover within the ED and the requirement to train new staff were barriers to the development of skills (2). To underpin screening, understanding medical equipment, screening and measurement methods and being able to critically evaluate results were viewed as vital skills (19, 26), for example, where an ECG (2) or blood results (22) are part of the screening process. In addition, staff had to use their skill in appropriately undertaking screening (16, 19, 21). They used their clinical judgement to decide if patients were well enough to be screened e.g. if a patient was distressed or critically unwell or if it was best to screen at a more appropriate time (2, 16)

ED nurses were described as highly skilled at the screening and referral process (2, 3, 6). This group easily identified those at risk through a combination of practical experience, clinical judgment and communication skills (2, 3, 6). For example, nurses used the patients’ appearance, extremes of age and comorbidities to determine whether a patient was high risk and required screening/referral (2, 15). Triage nurses, in particular, easily identified patient risk factors through conversation with patients and families and analysis of documentation and electronic sources of information (2, 3, 6). This approach limited the impact on the patient (3). The importance of HCWs being able to identify and integrate screening as a normal function of their role and not become overwhelmed by the additional workload that can be attributed to screening was identified (14, 28).

However, stressors within the ED such as high work volume or ill patients prevented holistic assessment and directed resources away from the recognition and response to patients who require screening and referral (3, 6, 9).

**Domain:** Skills

**Finding 4:** Recognising and responding to patients who required screening was challenging and dependent on appropriately trained and skilled clinicians/practitioners.

Staff skills development was essential to the adoption of screening (16, 18, 21). Many ED staff members were unwilling to engage in screening if they lacked to skills to do so safely (16, 21, 22). For example, staff felt uncomfortable when expected to use complex clinical algorithms (21) and/or screen for sepsis (22) without updating their skills. Recognising and responding to patients who required screening was challenging and dependent on appropriately trained and skilled physicians (9). HCWs did not always have the specific skills or experience required to identify patients who were eligible for screening and referral to appropriate pathways (1, 2, 9, 22). This was illustrated where junior registrars struggled to differentiate between a possible myocardial infarction and pulmonary embolus (2). Senior staff described inappropriate allocation of patients and were concerned that more junior staff members lacked the diagnostic skills to screen and refer patients effectively (1, 2). To screen and refer patients safely, ED staff required competence in patient assessment skills to inform the collection of a patient history and conduct physical assessments (13, 16). This was vital when screening patients with co-morbidities and complicated histories (e.g. Geriatric screening for adverse outcomes) (13, 16). Using these screening pathways and protocols reduced the need for clinical decision-making and could impact on the development of competent diagnostic skills among junior staff (1, 2, 22).

**Domains:** Emotions and Optimism

**Finding 5:** HCWs described how the implementation of screening processes in the ED impacted on their role and the care that they provided, staff experienced a variety of emotions when attempting to understand and deal with this impact including optimism, pessimism, fear, stress and a generalised negative affect.

HCWs expressed both optimistic and pessimistic outlooks for the implementation of screening in the ED. Multiple forms of screening were utilised within the ED and underpinned by guidelines, protocols or integrated as part of pathways. Consequently, HCWs were required to read and comprehend related documentation in a stressful, high-pressure environment (10, 23). Staff also viewed the volume and continuous addition and updating of guidelines negatively increasing the difficulty of adherence (10, 23). *I find the large number of guidelines totally stressing and it is completely unrealistic . . . especially because many of them (the guidelines) are not relevant for the staff in the emergency department. I wonder what the capital thinks we are spending our time doing* (Kirk & Nilsen, 2015 Pg561)**.** However, HCWs recognised the benefits of screening programmes for the patients, hospital and wider community (13, 16, 21, 23). Staff experienced that once screened and educated about the risk of illness e.g. hypertension, patients were far more likely to follow-up and engage with treatment (16, 19). *“I think this process will also result in compliance...I think someone who understands and recognizes the importance of normalizing their blood pressure will comply.”*  (Pirotte et al, 2014 e4). In addition, where staff were involved in the implementation process, they were more enthusiastic about their use and more optimistic of a successful outcome (16, 19). Organisational investment, in the form of funding and resources to support the screening and referral process, also enhanced staff optimism (21, 23). Furthermore, an optimistic perspective was associated with compliance to screening among staff (22, 23). (Example)Staff experienced improvements in compliance when strategic interventions to improve adherence such as tailored educational programmes were put in place and this was encouraging (10, 13, 16, 19, 21, 22, 23, 27).

HCWs experienced stress when screening patients with comorbidities (4,8,10). Staff felt that the process was difficult and required more time, judgment and in-depth knowledge of the patient to ensure accuracy (4,10). This was difficult to achieve in a busy ED. Communication barrier also caused anxiety where confused or cognitively impaired patients were perceived as difficult to screen (8). In some instances, patients could not provide the required information, and this was challenging (8). “*Professionals initially tried to talk directly to the patient, but in some circumstances, this was not possible, preventing the identification of some possible causes and consequences of falls, and ways in which to prevent re-occurrence”* (McEwan et al, 2018 Pg 4). In addition, ED nurses reported feeling a considerable burden when pressure from senior staff to meet patient flow targets induced anxiety (8, 10). Furthermore, the culture within the ED caused conflict where more experienced ED staff disapproved of “*newcomers*”, junior doctors and nurses who adhered to screening processes (8,22). Where senior staff favoured flow culture, those who engaged in screening were perceived as delaying patient flow (8, 22). New staff felt significant pressure to conform as a result and experienced significant stress (8,22). *We’ll quickly teach the new nurses that nutrition screening doesn’t* *belong in this department* (medical secretary 1 ED, interview) (Kirk & Nilsen, 2015 Pg561)**.** They also worried about neglecting to screen patients due to time constraints and forgetting to screen when indicated was also a source of stress (14, 26). Staff felt that structured documentation and templates could alleviate stress by ensuring that staff adhered to a uniform process (14, 26). *“Documentation templates reduce anxiety about forgetting, it makes sure that everything gets done and that we all do it the same way. It will decrease anxiety about forgetting*” (ED Doctor) (Skyttberg et al, 2016 Pg 5)

HCWs were fearful of having less time to spend with patients in order to screen, this included less time to spend with those who were critically ill (8,10,14). This could lead to an emotional burden on nurses where screening was then perceived as a negative aspect of their role (8, 10, 14). Non-adherence to evidence-based practices generated a guilty conscience. The nurses knew that the screening for nutrition was of relevance to the patients: *I have learned the importance of well-nourished patients, but there* *are other actions I have to prioritise in this department* (nurse 17, interview). (Kirk & Nilsen, 2015 Pg561).

HCWs experienced uncertainty where staff felt unsure about the screening process and found it difficult to make decisions. ED staff found it difficult to determine if or when care should be escalated based on screening results (10, 25). ‘*I’m never 100%, you know, ‘here’s my best guess of your chances,’ which if I’m talking to them, it’s usually a few percentage points, and I talk to them a bit about what they feel comfortable with in terms of* *discharge and treatment’* (Ref). Shared decision making was reported to mitigate this uncertainty and may involve the MDT and the patient (10, 25). “*Well, with the usual tools that we all have available to us… the risk assessment tools, the current one that I like to use more than the others is the HEART score, those current tools are good for medical legal documentation…I don’t really need the tools, but… I can at least say ‘this is what the predictor says*’ (Schoenfeld et al, 2019 Pg349). However, further clarity around the screening and referral process was needed to address this challenge and reassure staff (10, 25).

**Domain:** Motivation and Goals**,** Beliefs about Consequences

**Finding 6:** HCWs motivation to screen and goals for screening were influenced by a number of factors, this included the organisational culture in the ED, environmental stressors and ED staffs personal and professional motivations.

To inform implementation strategies, a clear understanding of HCWs motivations around screening is required (2,3,5,11,19,23). Staff were motivated when clear benefits for the patient and practice setting were indicated or identified (2,5,11,19). A reduction in the rates of readmission, the limitation of adverse outcomes and enhancement of patient safety provided the motivation to consistently engage in screening (2,3,5,11,19,23). ED staff were primarily motivated by the assurance and enhancement of patient safety and the collective achievement of positive patient outcomes (2,3,11,23). “*Several sites emphasized the benefit of the clinical protocols in enabling a more uniform standard of care for CDU patients, providing clear goals and expectations for patient care and improving patient safety: “...the first group of improvements involves the standardization of care these patients are receiving...[Previously, it was very practitioner-dependent...”* (Salkeld et al, 2011, Pg 367).

Furthermore, senior staff (doctors and nurses), management and organisations employing implementation strategies needed to remain cognisant of ED staffs’ intentions for implementation. Staff who were well informed about the benefits of screening were motivated to become involved in implementation strategies (2,3,10,11,12). A clear focus on the integration of screening in a busy ED environment and a clear rationale around the methods of implementation such as training and practice development to underpin screening were key to attaining staff buy-in (2,3,10,11,12). Staff wanted to be involved and engaged in the implementation process as they felt this ensured an easy to use and acceptable process. Consequently, this involvement limited the impact on staff and the ED setting as they were consistently orientated to the changes being made (3,8,21). As a result, education and changes to existing practices were required (3,8,21).

**Domain:** Motivation and Goals

**Finding 7:** Clear goals to implement screening were outlined and developed collaboratively with ED staff. These goals involved the establishment of “*preconditions*” for successful implementation where, when certain conditions are met, they could facilitate the process. These preconditions indicated a certainty and stability of intentions around implementation strategies and included organisational supports and multidisciplinary collaboration.

HCWs described needing reassurance around the rationale for screening and this could be achieved by orientating staff to the screening process prior to implementation (2, 11,16,19,23). Belief in successful implementation was dependent on staff making sense of the screening process and having clear goals (10, 11, 12, 19, 27). Screening must bring additional meaning and purpose to their role by providing new information about the patient and, consequently, identifying those at risk (10, 11, 12, 19, 27). Evidence to underpin the success of the screening process and illustrate the predictive value of the screening tool was also requested by ED staff (10, 11, 12). Reassuring and orientating staff was a clear goal or precondition for the successful implementation of screening.

Organisational supports such as study days, educational programmes, mentorship and designated staff members to support the screening process on-site were also identified by HCWs as preconditions for screening implementation (2,3,10,11,19,24). ED nurses described failing in the implementation of guidelines and screening tools when they had to include them in their daily practice with no additional resources (2,6,11,16, 27,28). For example, staff consistently described feeling enthusiastic and motivated to learn when supported by their organisation (2,4,10,11, 27). The co-ordination of workflow and systems management at an organisational level to support screening was also viewed positively (1,11,27,28,30). Meaningful efforts to assist staff in developing knowledge were perceived as positive and a facilitator of staff uptake (2,4,10,11). Staff hoped for organisational recognition of barriers to screening such as interruptions and operational failures (1,11,27,28,30). Implementation of the screening tools meant less time for other tasks and required leadership and understanding from management to support required adaptations and contingencies (2,6,28). Establishing contingencies to address these issues could form the basis for goal-setting prior to implementation (1, 27).

Multidisciplinary collaboration was also viewed by staff as a precondition for implementation where clear roles and responsibilities around screening were outlined (1,2,11,27,28). When this was not achieved, a variable approach to the process was perceived where team members were unsure of who was involved in the process and who was responsible when additional care and referral was required (1,2,11,27,28).

HCWs perceived that the ED environment impacted on screening goals (1,2,3,19,24). The ED screening was not always deemed a priority when staff could not align this process with the presenting complaint (2, 19,24) e.g. nutritional screening when the patient presents with chest pain. Therefore, screening goals were often perceived as a peripheral priority while staff focused on the specific illness the patient presented with (1,2,3,19,24). Screening tools were used inappropriately where the perceived need for more urgent patient care or referral to specialist services was facilitated by altering scores and attaining referrals and additional assessment based on these results (2,3,5,10,23). This created conflict between what staff perceive as the goal of screening and what the actual process was aiming to achieve e.g. detection of pain vs using the pain score to escalate care.

To overcome these challenges, setting clear goals and objectives to ensure a uniform screening process within the ED was perceived as positive by staff (2,6, 19, 23, 24). This ensured that practitioner interpretation was moderated, and implementation strategies were informed by staff experience (2,6, 19, 23, 24). This was achieved using evidence based clinical protocols and pathways which integrated the screening process and enabled a more uniform and consistent standard of care (2,3,23). To align staff goals with that of a newly implemented process or pathway the clear communication of screening goals was vital (2,3,23). A clear rationale pertaining to the patients being targeted and consistent clarity on the process is required to ensure staff buy-in (2,3,23).

Treatment targets within the ED also impacted on goal setting. ED staff perceived that the overall focus was on maintaining a flow culture (1,2,8,11). If patients were at risk of being outside a 4-hour treatment targets, screening was often omitted or altered (8). Maintaining a balance between treatment targets and appropriate screening was viewed by staff as essential to reduce staff sickness and to retain a highly motivated workforce (1,8).

**Domain:** Beliefs about Capabilities, Social Influences, Professional Role and Responsibilities

**Finding 8:** HCWs professional confidence impacted on the screening process, staff who felt empowered to screen facilitated the process.

HCW’s were empowered to screen through distributed leadership and education. HCWs felt empowered through screening as it resulted in a more appropriate and efficient use of the ED and a collaborative team-based approach to achieving all components of the screening and referral process (13, 15, 22). Screening tools and pathways helped to improve HCWs confidence and gave structure to the schedule of diagnostics, interventions and flow through the ED (5, 8 10). This was deemed vital as if HCWs lost confidence in their ability, they were less likely to seek out advice from more experienced team members and possibly omit screening (8, 16). It also gave them an evidence-based rationale for the treatment they provided (2,5,8,10). For particular presentations like chest pain, where staff found it difficult to make decisions or plan care, screening and assessment protocols gave staff confidence and empowered their decision-making (2, 8). “*It’s easy to use and it cuts out any indecision. If my seniors and consultants and registrar are using the pathway then I feel empowered, I guess, and confident that they have put their confidence in the pathway so that I should be able to as well*” (Crilly et al, 2020 P154). A team-based collaborative decision-making process was also favoured by staff who lacked confidence (2, 16, 22, 28). *“I wouldn’t make that part of my triage because I don’t have confidence in my entire grasp of it. And also, I think it needs to be something led from a team perspective*” (Tavender et al, 2014, Pg149). They also gave HCWs the confidence to communicate treatment and management concerns for vulnerable patients (5). Even after training and support, some HCWs felt they needed more time to develop competence and skills (16, 22, 28). Staff feared missing a diagnosis if clinical judgment was not factored in or they lacked experience (1, 2, 28). Screening patients who were intoxicated, cognitively impaired or combative was also a concern for ED doctors (28). *“...it’s usually quite straightforward in the straightforward patient; but if it’s someone with dementia or something everything is difficult.”* (Table S1 to S4 Tavender et al, 2014). Nurses felt empowered by screening pathways as they enhanced their autonomy within the ED (2, 5, 8, 18) *“… created autonomy as nurses because since the pathway has been brought out, if we think they are a very low-risk pathway then we won’t initiate blood work on them. But if they meet the intermediate- or high-risk pathway then we, as nurses, can initiate the measurement of the troponin and the cut off markers, so. I think it’s given us, if anything, a little bit more autonomy*” (Crilly et al, 2020 Pg 152). These screening pathways and protocols provided ED nurses with a more systematic way of communicating information to ED physicians and to the patient (5). The information they provided was also more objective (5).

**Domains:** Reinforcement, Environmental Context and Resources, Skills

**Finding 9:** HCWs experience indicates that the likelihood of a successful screening/referral process was dependent on a number of factors including rewards, incentives, contingency planning, reinforcement and sanctions.

HCWs showed appreciation for reward-based strategies for the implementation of screening and responded to positive feedback (2, 27), There was also a strong focus on motivating staff to achieve targets of completing the Sepsis Six within an hour, and rewarding them when they did so: the use of **“***It’s audited […] on a weekly or monthly basis by one of the registrars. So they come back to us and, with a chart to show us how well we’re doing. […] It makes you feel quite good actually when you know you’re up there [i.e. have high compliance with the Sepsis Six]* (Interview, senior nurse, Site 1) (Tarrant et al, 2016 Pg 3). However, being rewarded was not viewed as important to staff, they were more concerned with performing well and achieving screening goals and providing quality care (14, 22). *“The pleasure reported by nurses who perform risk assessment and classification is related to the minimization of the patient’s suffering and also assistance for the improvement of the clinical picture”* (Midori Sakai et al, 2016 Pg 237).

Approaches to implement screening tended to focus on individual behaviour and used behaviour change approaches focused on motivating, equipping, and empowering staff to adhere to best practice (13, 27). This included awareness raising, education, modelling, persuasion, reminders and prompts, and feedback (13, 27). The screening/referral process was incentivised by hospital management and ED managers by emphasising the potential benefits among ED staff (13, 14, 19, 22, 27). Where the success of a medical screening programme could result in a more efficient allocation of patients the potential to expand staffing and provider capacity was appealing to staff (13). *“Some appealing aspects of this program for RHC buy-in included the ability to expand staffing and provider capacity, and establish a new medical home for converted patients and their families or others referred by them*” (Menser et al, 2015 Pg 131). Furthermore, ED staff noted screening and referral programs could function as advocacy tools and encourage investment in resources for high impact evidence-based investigations and treatments (19, 21). Screening programmes that fell within ED staffs’ current scope of practice, without upskilling, was an incentive (19).

HCWs put contingencies in place to manage the impact of the screening and referral process (13, 14, 19, 23). For example, where a medical screening programme was implemented to refer low risk patients to minor injury clinics, increased demand for this service was expected and clinic hours were increased (13).

HCWs described methods of reinforcing the screening process (13, 19, 23, 27). ED staff emphasised the importance of maintaining control by setting clear screening expectations in the ED (23). This was achieved by increasing awareness among staff via system based electronic reminders and prompts on computer-based programmes (e.g. over triage and ED databases), posters and pathways present in documentation form for all staff to access was also useful to reinforce the uptake and usage of screening (13, 19, 27, 28). Ensuring inter-departmental transfer of records increased efficiency and avoided repeated handover of information between MDT members (13, 23). Assessing HCWs screening skills was also viewed as a method of reinforcing the process in the ED. However, the assessment of HCWs competence at screening was infrequently conducted (24, 26, 29). Multiple performance monitoring activities to assess ED staffs’ screening/referral skills were reported (18, 22, 28). This included explicit methods of providing regular constructive feedback on ED staff performance e.g. via staff meetings and performance reviews (22).

HCWs suggested that sanctions may be needed for staff who were reluctant to use screening tools and algorithms (8, 21, 22). Some ED staff did not easily take to screening and referral protocols, as a result, they were required to sign documentation stating they had completed aspects of the process at different intervals (21). This enabled management and senior staff to monitor adherence and follow-up with staff who were involved in the process (8, 21, 22). Nurses reported consistent pressure from monitoring of their performance, such as reporting to senior nurses on the ward or phone calls from managers (8). Where screening protocols, pathways and frameworks were not formally implemented in the workplace via departmental policy, they were not accepted as a method of assessment (27).

**Domain:** Memory, Attention and Decision-Making Processes, Environmental Context and Resources

**Finding 10:** HCWs memory and cognitive and decision-making processes impacted on their ability to screen and refer patients in the ED.

HCWs found some screening guidelines, frameworks and protocols difficult to memorise in a busy ED environment (2, 16, 21, 28). For example, screening patients for risk of myocardial infarction (2) and adverse outcomes with mTBI (Mild Traumatic Brain Injury) (28) was a complex process involving numerous criteria and assessments. ED staff found this challenging as they were expected to screen and refer patients promptly, in adherence to complex guidelines (2, 16, 21, 28). Therefore, staff favoured simple, clear screening tools and processes that were easy to remember and routinely used (21, 22, 26).

Staffs experience pin-pointed strategies that could assist with memory and recall. Evidence based clinical algorithms and screening and referral pathways presented as diagrams acted as memory aids when readily available to staff (2, 16, 21). Presenting these in poster form, throughout the ED, assisted with recall (e.g. screening criteria and risk factors) (2, 16, 21). For example, when risk stratifying patients with chest pain, nurses, residents and consultants were observed using the poster based representation of their screening, assessment and referral protocol to check if their risk assessment (Low, moderate, high risk for cardiac events) was accurate (2). Furthermore, easy access to an electronic repository where screening guidelines and tools were readily available to staff assisted to clarify or recall information (2, 21) Electronic health records that provided a digitised form of the screening referral process via checklists and portable pathways (e.g. on I-pads), ensured that everything was completed and no criteria were omitted (26)

ED staff prioritised requesting and attaining information from the patient and MDT team to inform their decision-making (2, 3). This included risk screening questions which pertained to their physical state, cognitive functioning and psychological well-being (3, 4, 6). In some instances, particularly in triage or during patient handovers, staff did not spend enough time attaining information to clarify patient's level of risk (2, 11, 24). Staff based their screening on brief communications with paramedics, what the patient looked like and what their vital observations indicated (24). In these instances, their decision-making lacked complete information (24). Family members assisted staff in decision making around risk (4, 5, 15). Where patients were cognitively impaired or poor historians, family members provided information that informed the screening process (4, 5, 15). Furthermore, HCWs described inconsistencies in how the screening and referral process was documented (11, 24, 26). Where some staff simply documented the screening results (score, or risk category), others described how they were attained. For example, by including patient reported risk factors, observation, physiologic and behaviour signs and co-morbidities (24, 26). A standardised process was perceived by staff to increase quality by reducing individual variations in documentation (26).

**Domain:** Intentions, Professional Role and Responsibilities, Memory, Attention and Decision-making Processes.

**Finding 11:** HCWs perform screening for several reasons including commitment to the patient and maintaining patient safety, they also resisted screening due to competing interests in the ED.

To implement screening and referral processes organisations must commit to increasing knowledge, raising awareness, motivating staff and restructuring the environment to facilitate the desired behaviour (8, 10, 19, 22). HCWs experience indicated that screening needs to be performed privately and in a dignified and respectful way (3, 28). Staff felt that this was important as it facilitated patient involvement by developing trust (3, 28). Furthermore, staff resolved to maintain patient safety, and this was achieved through patient education and involvement (3, 28). To fully inform the patient on the screening/referral process, staff required access to screening tools in different languages and written information (e.g. head injury advice) to provide the patient with on discharge (3, 28).

HCWs had clear intentions to develop their practice and used their free time to update their knowledge before new screening tools and algorithms were implemented (22, 27). ED nurses in particular felt responsible for delivering evidence-based care, and due to busy work environments often upskilled on days off (22, 27). HCWs chose to use these tools and algorithms as they improved their confidence, efficiency and improved the allocation of resources through systematic assessment (16, 27) For example, where evidence based clinical algorithms were used ED nurses felt that it made them deliver care promptly, avail of materials and human resources and stop unnecessary prescribing through informed decision-making (27).

HCWs described the screening process as more complicated than others may realise (19,22) Staff sometimes chose not to complete the screening process fully as the risks/benefits were different for each patient/situation (19, 22). In this instance, it was ED staffs’ intention to maintain patient safety by using their clinical judgment (22). This required critical thinking and a patient centred approach.

Furthermore, ED staff decided not to prioritise screening where they lacked the time to follow through with the screening/referral process (11, 22). While HCWs talked about a lack of time as a barrier to screening, spending time on tasks that did not support their professional role was a barrier to screening implementation (2, 11, 13, 19). For example, where staff routinely screened patients for hypertension in the ED, some staff consciously refused to engage in the process as they felt this was not part of their role and irrelevant if hypertension was not the patients presenting complaint (19).
