# Supplementary material for: A qualitative evidence synthesis (QES) exploring the barriers and facilitators to screening in emergency departments using the theoretical domains framework
Source: BMC Health Serv Res. 2023 Oct 11;23:1090. doi: 10.1186/s12913-023-10027-3 (PMC10568862; doi:10.1186/s12913-023-10027-3)
Supplement: Supplementary file 8 — Additional file 8: Supplementary file 8. Mapping of Barriers and Facilitators to Final Themes. [file 12913_2023_10027_MOESM8_ESM.docx]

| **Supplementary file 8 Mapping of Barriers and Facilitators to Final Themes:** Concepts from the TDF were then clustered and synthesised to form a final set of themes representing the whole dataset Therefore, after extracting data from studies pertaining to healthcare workers experience of screening in the ED, barriers and facilitators were identified from the constructs. This process is outlined in the table below. | | | | | |
| --- | --- | --- | --- | --- | --- |
| **Domain(s) and Linked Abbreviations** | **Finding** | **Articles that Informed the Finding** | **Identified Barriers Linked with Relevant Domains** | **Identified Facilitators Linked with Relevant Domains** | **Final Finding** |
| Knowledge **K**  Behavioural Regulation **BR** | 1. HCWs experience illustrates that procedural knowledge, and an awareness of knowledge deficits, can impact on the screening and referral process. | Abdulwahid et al (2018)  Crilly et al (2020)  Fry et al (2016)  Gwyther at al (2018)  Harley et al (2019)  McEwan et al (2018)  Menser et al (2015)  Munroe et al (2018)  Pirotte et al (2014)  Roberts et al (2017)  Salkeld et al (2011)  Schoenfeld et al (2019)  Tavender et al (2014)  Van Der Wulp et al (2011)  Wolf et al (2019) | Lack of procedural knowledge pertaining to the screening process (Policies, Guidelines, Contextual Relevance) **K**  Complicated screening procedures and pathways. **K, BR**  Perceived need to initiate or engage in education pertaining to screening process **BR** | Procedural Knowledge pertaining to the Screening Process **K**  In-depth, context specific knowledge pertaining to the screening process **K** | **Knowledge and Skills to Screen**  **Preconditions to Screen** |
| Knowledge **K** | 2. HCWs perceived lack of knowledge among ED staff and their peers pertaining to screening processed in the ED. | Abdulwahid et al (2018)  Crilly et al (2020)  Fry et al (2016)  Harley et al (2019)  McEwan et al (2018)  Roberts et al (2017)  Salkeld et al (2011)  Van Der Wulp et al (2011) | Lack of knowledge among staff pertaining to the rationale for screening, accurate and consistent use and the availability of validated tools **K**  Lack of standardisation of the screening process. **K** | Education tailored to address specific knowledge deficits and gain commitment to the process **K**  Easy access to resources to underpin and inform screening **K** | **Knowledge and Skills to Screen**  **Preconditions to Screen** |
| Skills **S**  Behavioural Regulation **BR** | 3. HCWs developed skills to engage in the screening and referral process competently, these skills were attained through practice-based experience, educational opportunities and skills assessment. | Crilly et al (2020)  Daniel et al (2015)  Fry et al (2016)  Gorawara-Bhat et al (2017)  Harley et al (2019)  Midori Sakai et al (2016)  Mistry et al (2016)  Munroe et al (2018)  Pirotte et al (2014)  Roberts et al (2017)  Salkeld et al (2011)  Van Der Wulp et al (2011) | Staff turnover within the ED. | Clear and accessible screening and referral processes facilitated screening skills development **S** | **Preconditions to Screen** |
| Skills **S** | 4. Recognising and responding to patients who required screening was challenging and dependent on appropriately trained and skilled clinicians/practitioners. | Abdulwahid et al (2018)  Crilly et al (2020)  Harley et al (2019)  Menser et al (2015)  Munroe et al (2018)  Olson et al (2011)  Pirotte et al (2014)  Roberts et al (2017)  Salkeld et al (2011) | Unwillingness to engage in screening if perceived lack of skills to do so safely **S**  Lack of skills to perform and respond to patients who required screening to access care/referrals **S** | Development of skills relevant to screening processes **S**  Staff may require competence in advanced assessment inclusive of history taking, interpretation of diagnostic tests and physical assessment to underpin the screening process **S** | **Knowledge and Skills to Screen** |
| Emotions and Optimism **EO** | 5.HCWs described how the implementation of screening processes in the ED impacted on their role and the care that they provided, staff experienced a variety of emotions when attempting to understand and deal with this impact including optimism, pessimism, fear, stress and a generalised negative effect. | Fry et al (2016)  Hoyle and Grant (2015)  Kirk and Nilsen (2015)  Menser et al (2015)  Midori-Sakai et al (2016)  Pirotte et al (2014)  Munroe et al (2018)  Roberts et al (2017)  Salkeld et al (2011)  Sampson et al (2019)  Skyttberg et al (2016)  Tarrant et al (2016)  Tavender et al (2014) | Competing interests in the ED and perception of screening as an additional stressor or workload **E**  Staff experienced fear and uncertainty that additional responsibility and decision making may impact on patient care and outcomes **E**  Non-adherence to embedded flow cultures and targets due to adherence to newly implemented screening practices resulted in resistance from staff **E**  Screening of patients with co-morbidities or cognitive impairment led to apprehension among staff **E** | Commitment to providing evidence-based patient care **O**  Professional motivation to enhance skills and deliver high quality patient care **O**  Structured documentation and templates to underpin the screening process were perceived positively **O**  Perceived positive impact of screening on patient care provision **O**  Organisational investment in resources that underpin and facilitate screening was perceived positively and viewed as vital **O** | **Motivation to Screen** |
| Motivation and Goals **MG**  Beliefs about Consequences **BC** | 6. HCWs motivation to screen and goals for screening were influenced by several factors, this included the organisational culture in the ED, environmental stressors and ED staffs personal and professional motivations. | Crilly et al (2020)  Daniel et al (2015)  Fry et al (2016)  Hoyle and Grant (2015)  Kirk and Nilsen (2015)  Kirk et al (2016)  McEwan et al (2018)  Pirotte et al (2014)  Sampson et al (2019) | Lack of understanding or input in the implementation of screening processes **M** | HCWs were motivated to screen where a positive impact on patient care could be articulated, enhancing patient safety or reducing readmission **MG BC**  Organisational and managerial awareness and planning must be based on ED staffs’ intentions and input pertaining to the implementation of screening (Buy-in) **MG BC** | **Motivation to Screen**  **Preconditions to Screen**  **Knowledge and Skills to Screen.** |
| Motivation and Goals **MG** | 7. Clear goals to implement screening were outlined and developed collaboratively with ED staff. These goals involved the establishment of “*preconditions*” for successful implementation where, when certain conditions were met, they could facilitate the process. These preconditions indicated a certainty and stability of intentions around implementation strategies and included organisational supports and multidisciplinary collaboration. | Abdulwahid et al (2018)  Crilly et al (2020)  Daniel et al (2015)  Fry et al A (2016)  Gorawara-Bhat et al (2017)  Hoyle and Grant (2015)  Kirk and Nilsen (2015)  Kirk et al (2016)  McEwan et al (2016)  Munroe et al (2018)  Pirotte et al (2014)  Sampson et al (2019)  Schoenfeld et al (2019)  Tavender et al (2014)  Van der Wulp et al (2011)  Eagles et al (2022) | A lack of MDT collaboration can result in a variable approach to screening **MG**  A lack of resources to support staff in integrating screening particularly during initial implementation. **MG**  Lack of recognition, at an organisation level, of barriers that may impact on implementation including stressors in the ED and an increased workload. **MG**  Screening was not prioritised where the perceived needs of the patient did not align with the goals of the screening process. **MG** | Identifying preconditions to screening within each ED to facilitate implementation. **MG**  Identification of clear goals for implementation and orientation of staff.  **MG**  MDT collaboration where clear roles and responsibilities around screening were outlined. **MG**  Tailored education supports and the co-ordination of workflow and systems management at an organisational level to support screening. **MG**  Required adaptations and contingency planning to pre-empt issues that may arise during implementation. **MG** | **Preconditions to Screen**  **Motivation to Screen** |
| Beliefs about Capabilities **BAC**  Social Influences **SI**  Professional Roles and Responsibilities  **PRR** | 8. HCWs professional confidence impacted on the screening process, staff who felt empowered to screen facilitated the process. | Abdulwahid et al (2018)  Crilly et al (2020)  Fry et al B (2016)  Hoyle and Grant (2015)  Kirk and Nilsen (2015)  Menser et al (2015)  Mistry et al (2018)  Munroe et al (2018)  Olson et al (2011)  Salkeld et al (2011)  Van der Wulp et al (2011) | HCWs feared errors where clinical judgment, along with screening results, were not considered **BAC, PRR**  HCWs were apprehensive when screening patients who were cognitively impaired **BAC**  For some HCWs, even after training and support, they needed further support, leadership and guidance to develop competence and confidence in their screening practices **BAC, SI, PRR** | HCWs felt empowered through screening as it yielded more appropriate and collaborative approach to care provision and efficient use of ED and linked community resources **BAC**  Screening gave structure and an evidence-based rationale for treatment provided and consequently enhanced HCWs confidence and underpinned decision-making and communication of patient needs **BAC, PRR** | **Motivation to Screen**  **Knowledge and Skills to Screen.** |
| Reinforcement **R**  Environmental Context and Resources **ECR**  Skills **S** | 9. HCWs experience indicates that the likelihood of a successful screening/referral process was dependent on several factors including rewards, incentives, contingency planning, reinforcement and sanctions. | Crilly et al (2020)  Daniel et al (2015)  Fry et al A (2016)  Fry et al B (2016)  McEwan et al (2018)  Mistry et al (2018)  Munroe et al (2018)  Roberts et al (2017)  Salkeld et al (2011)  Tarrant et al (2016)  Van der Wulp et al (2011)  Eagles et al (2022) | Screening protocols, pathways and frameworks, that were not formally implemented in the workplace via departmental policy, may not be accepted by staff **R, ECR** | Reward-based strategies for the implementation of screening, constructive feedback and formal assessment of skills were facilitators identified by staff **R, ECR, S**  Behaviour change approaches focusing on motivating, equipping and empowering staff to adhere to evidence-based screening/referral practices. **R, ECR**  Pre-empting need for additional resources that may arise from the screening process. This may include additional staff or need for clinics **R, ECR, S**  Organisations and management setting clear goals for staff pertaining to screening and referral requirements in the ED. Including mandatory attendance at education sessions **R, ECR.**  Leadership within the ED to support staff during implementation **R, ECR, S.** | **Preconditions to Screen**  **Motivation to Screen**  **Knowledge and Skills to Screen** |
| Memory, Attention and Decision-Making Processes  **MAD**  Environmental Context and Resources  **ECR** | 10, HCWs perform screening for several reasons including commitment to the patient and maintaining patient safety, they also resisted screening due to competing interests in the ED. | Daniel et al (2015)  Hoyle and Grant (2015)  Kirk and Nilsen (2015)  Kirk et al (2016)  Menser et al (2015)  Munroe et al (2018)  Pirotte et al (2014)  Salkeld et al (2011)  Tavender et al (2014)  Van der Wulp et al (2011) | Difficulty memorising screening protocols, pathways and frameworks in a busy ED environment **ECR, MAD**  HCWs often did not have adequate time to attain patient information relevant to the screening process; patient history. Therefore, their decision-making lacked complete information **ECR, MAD** | Staff favoured simple, clear screening tools and processes that were easy to remember and routinely used **ECR**  Clinical algorithms and diagrams representative of screening and referral pathways were favoured by staff as a memory-aid **ECR, MAD**  Digitised screening documentation was also seen as a facilitator which saved time and aided recall **ECR, MAD**  Family members aided in provided information relevant to the screening process; falls history when screening for risk **MAD**  A standardised process of documenting the screening process was also favoured by staff to ensure consistency and communication of decision-making. | **Preconditions to Screen**  **Motivation to Screen**  **Knowledge and Skills to Screen** |
| Intentions **I**  Professional Role and Responsibilities  **PRR**  Memory, Attention and Decision-making Processes.  **MAD** | 11, HCWs perform screening for several reasons including commitment to the patient and maintaining patient safety, they also resisted screening due to competing interests in the ED. | Crilly et al (2020)  Daniel et al (2015)  Hoyle and Grant (2015)  Kirk and Nilsen (2015)  Kirk et al (2016)  Menser et al (2015)  Munroe et al (2018)  Pirotte et al (2014)  Salkeld et al (2011)  Tavender et al (2014)  Van der Wulp et al (2011) | HCWs perceived the screening process as complex and difficult to implement in the ED **MAD**  The busy ED environment did not facilitate a dignified method of screening **I**  If screening was not related to the presenting complaint, or what they perceived as a function of their role, some staff refused to engage in screening **PRR**  HCWs may not prioritise screening if they lacked time to follow through with the screening and referral process **I, MAD** | Organisations must commit to increasing knowledge, raising awareness, motivating staff and restructuring the ED environment to facilitate screening **I, MAD**  Screening needs to be preformed in a dignified manner in a private setting **I, MAD, PRR**  Screening needed to fully involve the patient, informing them of the process and outcome **I, MAD, PRR**  HCWs had clear intentions to develop their practice and used their free time to update their knowledge before new screening tools and algorithms were implemented **I, PRR** | **Motivation to Screen**  **Knowledge and Skills to Screen.** |
