# Supplementary material for: A qualitative evidence synthesis (QES) exploring the barriers and facilitators to screening in emergency departments using the theoretical domains framework
Source: BMC Health Serv Res. 2023 Oct 11;23:1090. doi: 10.1186/s12913-023-10027-3 (PMC10568862; doi:10.1186/s12913-023-10027-3)
Supplement: Supplementary file 9 — Additional file 9: Supplementary file 9. GRADE CERQual Confidence in Findings. [file 12913_2023_10027_MOESM9_ESM.docx]

| **Supplementary file 9 GRADE CERQual Confidence in Findings:** Upon CASP appraisal, no significant concerns were noted for 15 articles (High Quality), 10 articles were deemed as having minor methodological limitations, one study as having minor-moderate limitations and two as having moderate (Moderate Quality). Only two studies were deemed as having major limitations (Low Quality). Upon application of GRADE CERQual, a high level of confidence in research findings was attained with minor concerns pertaining to adequacy and methodological limitations overall. A large number of studies from diverse populations and settings with predominantly rich data informed review findings. | | | |
| --- | --- | --- | --- |
| **Summary of Review Finding** | **GRADE-CERQual Assessment of Confidence in the Evidence** | **Studies Contributing to Review Finding** | **Explanation of GRADE CERQual Assessment** |
| 1. HCWs experience illustrates that procedural knowledge and an awareness of knowledge deficits can impact on the screening and referral process. | High Confidence | 1,2,4,7,9,11, 13,16,21,22,24,27, 28, 29, 30 | Methodological Limitations: 2 Low 8 Moderate 7 High CASP Rating. Moderate concerns.    Minor concerns about coherence. Finding reflects the phenomenon of interest.  Minor concerns regarding adequacy. The data underlying the review finding is rich from a large number of studies which a diverse cohort of participants. 17 studies in total informed this finding.  No concerns regarding relevance. The primary research study contexts align with that of the overall review. With each study having direct relevance to the review question. |
| 2. HCWs perceived a lack of knowledge among ED staff and their peers pertaining to screening processes in the ED. | High Confidence | 1,2,5,9,12,21,22,28,30 | Methodological Limitations: 4 Moderate 5 High CASP Rating. Minor concerns.    Minor concerns about coherence. Finding reflects the phenomenon of interest. No significant concerns about the fit between the data from primary studies and the review finding.  Minor concerns regarding adequacy. The data underlying the review finding is rich from a large number of studies and a diverse cohort of participants. 9 studies in total informed this finding.  No concerns regarding relevance. The primary research study contexts align with that of the overall review. The findings were directly relevant to the review question. |
| 3. HCWs developed skills to engage in the screening and referral process competently, these skills were attained through practice based experience, educational opportunities and skills assessment. | High Confidence | 2,3,4,6,9,14,15,16,19,21,26,28,30 | Methodological Limitations: 4 Moderate 5 High CASP Rating. Minor concerns.    Minor concerns about coherence. Finding reflects the phenomenon of interest. No significant concerns about the fit between the data from primary studies and the review finding.  Minor concerns regarding adequacy. The data underlying the review finding is rich from a large number of studies and a diverse cohort of participants. 9 studies in total informed this finding.  No concerns regarding relevance. The primary research study contexts align with that of the overall review. The findings were directly relevant to the review question. |
| 4. Recognising and responding to patients who required screening was challenging and dependent on appropriately trained and skilled physicians. | Moderate Confidence | 1,2,7,9,13,16,18,21,22 | Methodological Limitations: 1 Low 3 Moderate 5 High CASP Rating. Moderate concerns.    Minor concerns about coherence. Finding reflects the phenomenon of interest. No significant concerns about the fit between the data from primary studies and the review finding.  Minor concerns regarding adequacy. The data underlying the review finding is rich from a large number of studies and a diverse cohort of participants and settings. Thin data from 2 studies with rich data from 7. 9 studies in total informed this finding.  No concerns regarding relevance. The primary research study contexts align with that of the overall review. The findings were directly relevant to the review question. |
| 5. HCWs described how the implementation of screening processes in the ED impacted on their role and the care that they provided, staff experienced a variety of emotions when attempting to understand and deal with this impact including optimism, pessimism, fear, stress and a generalised negative affect. | High Confidence | 4,8,10,13,14,16,19,21,22,23,25,27, 30 | Methodological Limitations: 1 Low 2 Moderate 10 High CASP Rating. Minor concerns.    Minor concerns about coherence. Finding reflects the phenomenon of interest. No significant concerns about the fit between the data from primary studies and the review finding.  Minor concerns regarding adequacy. The data underlying the review finding is rich from a large number of studies and a diverse cohort of participants. 13 studies in total informed this finding.  No concerns regarding relevance. The primary research study contexts align with that of the overall review. The findings were directly relevant to the review question. |
| 6. HCWs motivation to screen and goals for screening were influenced by a number of factors, this included the cultural climate in the ED, environmental stressors and ED staffs personal and professional motivations. | High Confidence | 2,3,5,8,10,11,12,19, 23, 30 | Methodological Limitations: 4 Moderate 6 High CASP Rating. Moderate concerns.    Minor concerns about coherence. Finding reflects the phenomenon of interest. No significant concerns about the fit between the data from primary studies and the review finding.  Minor concerns regarding adequacy. The data underlying the review finding is rich from a large number of studies and a diverse cohort of participants. 10 studies in total informed this finding.  No major concerns regarding relevance. The primary research study contexts align with that of the overall review. The findings were directly relevant to the review question. |
| 7. Clear goals to implement screening were outlined and formed collaboratively with ED staff. These goals involved the establishment of “*preconditions*” for successful implementation where, when certain conditions are met, they could facilitate the process. These preconditions indicated a certainty and stability of intentions around implementation strategies and included organisational supports and multidisciplinary collaboration. | High Confidence | 2,3,4,6,8,10,11,12,16,19,23,24,2728,30 | Methodological Limitations: 8 Moderate 7 High CASP Rating. Moderate concerns.    Minor concerns about coherence. Finding reflects the phenomenon of interest. No significant concerns about the fit between the data from primary studies and the review finding.  Minor concerns regarding adequacy. The data underlying the review finding is rich from a large number of studies and a diverse cohort of participants. 15 studies in total informed this finding.  No concerns regarding relevance. The primary research study contexts align with that of the overall review. The findings were directly relevant to the review question. |
| 8. HCWs professional confidence impacted on the screening process, staff who felt empowered to screen facilitated the process. | High Confidence | 2,5,8,10,13,15, 16, 18, 22,26,28. | Methodological Limitations: 5 Moderate 6 High CASP Ratings. Moderate concerns.    Minor concerns about coherence. Finding reflects the phenomenon of interest. No significant concerns about the fit between the data from primary studies and the review finding.  Minor concerns regarding adequacy. Predominantly, the data underlying the review finding is rich from a large number of studies and a diverse cohort of participants and countries. 11 studies in total informed this finding.  No concerns regarding relevance. Predominantly, the primary research study contexts align with that of the overall review. The findings were directly relevant to the review question. |
| 9. HCWs memory and cognitive and decision making processes impacted on their ability to screen and refer patients in the ED. | High Confidence | 2,3,4,5,12,15,16,21,22,26,28,30 | Methodological Limitations: 6 Moderate 6 High CASP Ratings. Moderate concerns.    Minor concerns about coherence. Overall, the findings reflect the phenomenon of interest. No significant concerns about the fit between the data from primary studies and the review finding.  Minor concerns regarding adequacy. Predominantly, the data underlying the review finding is rich, from a large number of studies and a diverse cohort of participants and countries. 12 studies in total informed this finding.  No concerns regarding relevance. Predominantly, the primary research study contexts align with that of the overall review. The findings were directly relevant to the review question. |
| 10, HCWs perform screening for a number of reasons including commitment to the patient and maintaining patient safety, they also resisted screening due to competing interests in the ED. | High Confidence | 3,8,10,11,13,16,19,22,27,28. | Methodological Limitations: 3 Moderate 8 High CASP Ratings. Minor concerns.    Minor concerns about coherence. Overall, the findings reflect the phenomenon of interest. No significant concerns about the fit between the data from primary studies and the review finding.  Minor concerns regarding adequacy. Predominantly, the data underlying the review finding is rich, from a large number of studies and a diverse cohort of participants and countries. 10 studies in total informed this finding.  No concerns regarding relevance. Predominantly, the primary research study contexts align with that of the overall review. The findings were directly relevant to the review question. |
| 11, HCWs perform screening for several reasons including commitment to the patient and maintaining patient safety, they also resisted screening due to competing interests in the ED. | High Confidence | 2, 3, 8, 10, 11, 13, 16, 19, 22, 27, 28. | Methodological Limitations: 4 Moderate 7 High CASP Ratings. Minor concerns.    Minor concerns about coherence. Overall, the findings reflect the phenomenon of interest. No significant concerns about the fit between the data from primary studies and the review finding.  Minor concerns regarding adequacy. Predominantly, the data underlying the review finding is rich, from a large number of studies and a diverse cohort of participants and countries. 11 studies in total informed this finding.  No concerns regarding relevance. Predominantly, the primary research study contexts align with that of the overall review. The findings were directly relevant to the review question. |
